# Identification of IL-27 as a novel regulator of major histocompatibility complex class I and class II expression, antigen presentation, and processing in intestinal epithelial cells

**DOI:** 10.3389/fimmu.2023.1226809

**Published:** 2023-09-25

**Authors:** Julia Diegelmann, Stephan Brand

**Affiliations:** ^1^ Department of Medicine II, Ludwig-Maximilians-Universität (LMU) University Hospital, LMU Munich, Munich, Germany; ^2^ Department of Conservative Dentistry and Periodontology, LMU University Hospital, LMU Munich, Munich, Germany; ^3^ Department of Gastroenterology and Hepatology, Kantonsspital St. Gallen, St. Gallen, Switzerland

**Keywords:** intestinal epithelial cells, IL-27, MHC receptor, antigen presentation, inflammatory bowel disease, intestinal inflammation, mucosal immunity, Crohn’s disease

## Abstract

Antigen presentation via major histocompatibility complex (MHC) class I and class II receptors plays a fundamental role in T cell-mediated adaptive immunity. A dysregulation of this fine-tuned recognition might result in the development of autoimmune diseases such as inflammatory bowel diseases that are characterized by chronic relapsing inflammation of the intestinal tract and a damaged intestinal epithelial barrier. While MHCII receptors are usually expressed by professional antigen presenting cells (APC) only, there is increasing evidence that non-immune cells such as intestinal epithelial cells (IEC) might express MHCII upon stimulation with IFN-γ and thus act as non-professional APC. However, little is known about other factors regulating intestinal epithelial MHC expression. Here, we identify IL-27 as an inducer of different MHCI and MHCII receptor subtypes and the invariant chain (CD74/li) in IEC via the STAT1/IRF1/CIITA axis. CIITA, MHCII, and CD74 expression was significantly increased in IEC from Crohn’s disease (CD) patients with active disease compared to controls or CD patients in remission. IEC phagocytosed and digested external antigens and apoptotic cells. IL-27 strongly stimulated antigen processing via the immunoproteasome in a IRF1-dependent manner. In co-culture experiments, antigen-primed IEC strongly enhanced lymphocyte proliferation and IL-2 secretion, dependent on direct cell-cell contact. IL-27 pretreatment of IEC significantly increased CD4^+^ T cell proliferation and reduced IL-2 levels in lymphocytes in coculture. In summary, we identified IL-27 as a novel regulator of IEC antigen processing and presentation via MHCI and MHCII receptors, underscoring the importance of IEC as non-professional APC.

## Introduction

The human immune defence is based on the ability of the innate and adaptive immune system to distinguish between self and non-self (foreign) antigens. If this fine-tuned recognition is dysregulated, autoimmune diseases, in which the immune system erroneously turns against self-antigens, might evolve. Inflammatory bowel diseases (IBD) are a group of autoimmune diseases that are characterized by chronic relapsing inflammation of the intestinal tract and a damaged intestinal epithelial barrier. IBD encompasses the two major forms Crohn’s disease (CD) and ulcerative colitis (UC). The pathogenesis of IBD is so far not fully elucidated, but it has been suggested that an exaggerated immune response in genetically susceptible individuals, together with the intestinal microbiota and other environmental factors, triggers and sustains the chronic inflammation (1).

In the complex differentiation between self and non-self-antigens, major histocompatibility complex (MHC) receptors, also known as human leukocyte antigen (HLA), play an essential role (2). They are expressed on the cell surface and present small antigenic peptides (self and non-self) to T cells, thereby inducing either immune tolerance or an inflammatory immune response.

MHC class I receptors are expressed on every nucleated cell although absolute levels between different cell types might vary strongly (2). They generally present peptides derived from intracellular proteins (both cellular proteins and foreign peptides, e.g., virus particles) to cytotoxic CD8+ T cells (2). However, MHCI-mediated cross-presentation of exogenous-derived antigens to CD8^+^ T cells has been described (3).

Peptides presented by MHCI receptors are generated by digestion of intracellular proteins in a specialized proteasome called immunoproteasome. Upon stimulation with IFN-γ, three subunits (β1c, β2c, β5c) of the catalytic core of the constitutive 20S proteasome are replaced by the specialized immunoproteasome subunits β1i (=PSMB8/LMP-7), β2i (=PSMB9/LMP-2), and β5i (=PSMB10/MECL-1) (4). Moreover, the catalytic activity of the 20S immunoproteasome can be further enhanced by the heptameric proteasome activator complex PA28 (4). Peptides from extracellular antigens (such as bacteria) are presented via MHC class II receptors to CD4^+^ T helper (Th) cells (2). These peptides are usually derived from endocytosed proteins that are digested by lysosomal proteases. In contrast to the ubiquitously expressed MHCI receptors, MHCII receptors are mainly found on professional antigen presenting cells (APC) such as macrophages or dendritic cells (2). However, other cell types such as fibroblasts (5) or endothelial cells (6) may act as non-professional APCs expressing MHCII on their surface upon stimulation with IFN-γ.

There is increasing evidence that intestinal epithelial cells (IEC) might serve as non-professional APCs as well. IEC form a heterogeneous single cell barrier separating host tissues from luminal bacteria and food antigens in the gut. Their constant contact with antigens requires a fine-tuned, adaptable response to establish oral tolerance and to maintain a physiological steady-state of low inflammation. However, the role of IEC in antigen presentation and intestinal immunity including IBD is only beginning to be defined.

While constitutive but variable expression of MHCII has been shown in IEC of the small intestine along the crypt-villous and the cranial-caudal axis, colonic IEC do not express MHCII under steady-state conditions (7, 8). The low constitutive MHCII levels in IEC are markedly increased under inflammatory conditions *in vitro* (IFN-γ treatment) and in IBD patients *in vivo* (7). Moreover, increased expression of costimulatory molecules (such as CD40, ICOSLG, PD-L1) has been observed in IEC of IBD patients (9, 10).

The expression of MHCI and MHCII genes essentially depends on the presence of two transcriptional regulators, namely interferon regulatory factor 1 (IRF1) and class II transactivator (CIITA). IRF1 binds to the interferon stimulated response element (ISRE) in the promoter of MHCI genes (11). CIITA is the master regulator of MHCII gene expression (12) and acts as transcriptional coactivator that does not bind DNA itself, but helps to assemble the transcription factor complex necessary for MHCII transcription (13).

Both transcription factors might be upregulated by proinflammatory stimuli, especially IFN-γ playing a major role (12, 14). While the function of IFN-γ in inducing MHC gene expression is well characterized, little is known about other factors regulating MHC expression in non-immune cells. Interestingly, it has been demonstrated that the cytokine IL-27 exerts an IFN-γ-like STAT1-driven response on human hepatocytes by inducing similar gene expression patterns including IRF1 (15). Moreover, there is evidence that IL-27 might up-regulate MHC expression in endothelial cells and monocytes (16, 17). IL-27 is a pleiotropic cytokine with pro- and anti-inflammatory properties (18) that was originally identified as a product of activated APC (19). However, very recent studies identified IEC and intestinal Tregs as an important local source of IL-27 (20, 21).

In a previous study, we showed for the first time that IEC express the IL-27 receptor complex and are responsive to IL-27 (22). In inflamed tissue of IBD patients, we measured elevated levels of IL-27, and an increased expression of its receptor subunits IL-27RA and gp130 in IEC (22). Single nucleotide polymorphisms (SNPs) in the *IL27* gene had been linked to IBD susceptibility by us and others (23, 24). In a microarray analysis, we identified CIITA as a major IL-27 target gene in IEC and also identified IRF-1 as IL-27-induced gene (22).

Given that IL-27-induced IRF1 and CIITA are known master regulators of MHC class I and class II gene transcription, respectively, and that the expression of IL-27 and its receptors is increased in IBD, we aimed to analyze here in detail the so far unknown functional role of IL-27 in IEC-mediated antigen presentation via MHC receptors, including a detailed analysis of involved IL-27 signaling pathways in IEC.

## Materials and methods

### Reagents and antibodies

Recombinant human IL-27, IFN-γ, and TNF-α were from R&D Systems (Wiesbaden, Germany). Small inhibitory (si) RNAs were from Ambion (Applied Biosystems, Darmstadt, Germany). PCR primers and biotin-labeled probes for gelshift assays were synthesized by TIB Molbiol (Berlin, Germany). For details on antibodies, fluorescent dyes, and labeled substrates, see **Supplemental Data**.

### Cell culture

Human intestinal epithelial cell lines DLD-1 and HT-29 cells (LGC Standards, Wesel, Germany) were grown in Dulbecco’s modified Eagle medium (DMEM) with 10% fetal bovine serum (FBS) and 5% penicillin/streptomycin in a humidified 5% CO_2_ atmosphere at 37°C.

Primary colonic epithelial cells (Sciencell, San Diego, U.S.A.) were cultured on poly-L-lysine-coated cell culture dishes in serum-free Colonic Epithelial Cell Medium (Sciencell) with Colonic Epithelial Cell Growth Supplement (Sciencell).

### RNA isolation and reverse transcription

Total RNA was isolated using the RNeasy Mini Kit from Qiagen (Hilden, Germany) according to the manufacturer’s instructions. RNA concentration and purity was determined photometrically. 500 ng RNA were reverse transcribed with the Transcriptor First Strand cDNA Synthesis Kit (Roche, Mannheim, Germany) using oligo d(T) primers in a total volume of 20 µl.

### Quantitative PCR

Real-time qPCR (for primer sequences see **Supplemental Table 1**) was performed on a LightCycler480 instrument with SYBR Green PCR Master Mix from Roche using 3 µM of each primer. All primers were designed not to amplify genomic DNA. Gene expression was calculated using the ΔΔC_T_ method. For details, see **Supplemental Methods**.

### siRNA transfection

DLD-1 cells were reversed transfected using Lipofectamine RNAiMAX (Life Technologies/Invitrogen, Darmstadt, Germany). A typical transfection experiment included 10 nM siRNA and 1 µl of Lipofectamine RNAiMAX per 1x10^5^ cells. Knockdown of the respective genes was determined 24 hours and 48 hours post transfection by qPCR and western blot, respectively. IL-27 stimulation was conducted 48 hours post transfection.

### Protein isolation and immunoblotting

Total protein was isolated by lysing the cells in RIPA buffer for 20 minutes on ice and clearing the supernatant by centrifugation. Protein concentration was determined by BCA Assay (ThermoFisher Scientific). Polyacrylamide gel electrophoresis and immunoblotting was performed according to standard procedures (25).

### Nuclear extracts and electrophoretic mobility shift assay (EMSA)

Nuclear extracts were isolated and EMSA was performed as described in the **Supplemental Methods**.

### Biotin labeling of surface proteins

Proteins expressed on the cell surface were biotin-labeled with the Pierce Cell Surface Protein Biotinylation and Isolation Kit (ThermoFisher Scientific) as specified in the **Supplemental Methods**.

### Immunoproteasome activity assay

IP activity was measured using short, IP subunit-specific amino acid sequences coupled to a fluorophore whose fluorescence is quenched but can be detected when the substrate is cleaved by the IP activity of cellular extracts. For details, see **Supplemental Methods**.

### Cell labeling with ViaFluor-488 SE and PKH26 fluorescent dyes

DLD-1 cells were labeled with ViaFluor-488 SE, a dye that is initially non-fluorescent and diffuses passively into live cells where it is converted to fluorescent dye by intracellular esterases. HT-29 cells were stained with the red fluorescent dye PKH26, (see **Supplemental Methods** for staining procedure details).

### Apoptosis and necrosis induction and phagocytosis assay

Apoptotic cell death of PKH26-labeled HT29 cells was induced by combined treatment with 20 ng/ml TNF-α (T) and 50 nM of the Smac mimetic SM-164 (S) as described (26). Necroptotic cell death was induced by combined treatment of T and S and 20 µM caspase inhibitor Z-VAD-fmk (Z) as described (26) for 24 hours. Necrosis was induced by treatment with 0.01% Triton X-100 or by a freeze-thaw procedure. After 24 hours, floating (dead) cells were collected by centrifugation and counted. They were added to DLD-1 cells (seeded on microscope slides and labeled with ViaFluor-488 SE beforehand) at a ratio of 4:1. After 24 hours of coculture, slides were washed with PBS and fixed with 4% paraformaldehyde. The nucleus was counterstained with ToPro3. Cells were analyzed by confocal laser scanning microscopy on a Zeiss LSM510 confocal laser scanning microscope.

### Immunofluorescence staining

DLD-1 cells were seeded on microscope chamber slides and were allowed to attach overnight. After stimulation with IL-27 for 48 hours, cells were fixed with ice-cold methanol and immunofluorescence was performed essentially as described previously (27).

### Human biopsy sampling and immunohistochemistry

All participating subjects gave written, informed consent prior to biopsy sampling. The study was approved by the Ethics committee of the Ludwig-Maximilians-University Munich (Department of Medicine) and adhered to the ethical principles for medical research involving human subjects of the Helsinki Declaration. The diagnosis of CD was based on endoscopic, radiological, and histopathological parameters, adhering to established international guidelines (28). For analysis, active CD was defined as Crohn’s disease activity index (CDAI) >150; remission of disease was defined as CDAI < 150. Colonic biopsies were taken from CD patients and control patients without a history of IBD undergoing colonoscopy and were embedded in paraffin. Slides with paraffin-embedded biopsies were stained as described (29) (for details, see **Supplemental Methods**).

### Peripheral blood mononuclear cells and CD4^+^ T cell isolation

PBMC were isolated by density gradient centrifugation as described in the **Supplemental Methods**. For the enrichment of CD4^+^ T cells, blood samples were mixed with RosetteSep CD4^+^ T cell enrichment cocktail (Stemcell Technologies) prior to centrifugation.

### Direct and indirect coculture of IEC and CD4^+^ T cells or PBMC

DLD-1 cells were preincubated with or without 50 ng/ml IL-27 for 24 hours to induce MHC receptor expression. Then, 30 ng/ml Staphylococcus enterotoxin A (SEA) or B (SEB) (Sigma Adrich), respectively, were added and the cells were incubated for another 48 hours to allow binding, uptake, and processing of the respective antigens. After a total time of 72 hours, 4 µg/ml mitomycin C were added to inhibit DNA synthesis and thereby to reduce BrdU incorporation into DLD-1 cells. After 2 hours, the cells were washed extensively with PBS to remove remaining cytokine, antigens, and mitomycin C. Cells were harvested by trypsination and counted. For direct coculture experiments, they were seeded in 96 well plates or 12 well plates together with freshly isolated PBMC or CD4^+^ T cells at a cell number ratio of 1:5. Control wells contained either only DLD-1 cells or only PBMCs/CD4^+^ T cells.

For indirect coculture, DLD-1 cells were seeded on the bottom of a 12 well plate while PBMC or CD4^+^ T cells were added in Transwell inserts (0.4 µm pore size) to prevent direct cell-cell contact. Cells were cultured between 48 and 72 hours before cell proliferation was determined using the colorimetric BrdU cell proliferation ELISA (Roche). For further details, see **Supplemental Methods**.

### IL-2 enzyme-linked immunosorbent assay

IL-2 levels from coculture supernatants were determined with the BD OptEIA™ Human IL-2 ELISA Kit II (BD Biosciences) according to the manufacturer’s instructions. All samples and standards were run in duplicates.

### Statistical analysis

Data analysis was performed with the SPSS software (IBM SPSS Statistics Version 27, International Business Machines Corporation, NY, U.S.A.). Data were analyzed by one-way ANOVA followed by Tukey’s-HSD *post-hoc* test for equal distributed variances and Games-Howell post–hoc test for unequal distributed variances. The statistical analysis was conducted at 95% confidence level. A p value of less than 0.05 was considered as statistically significant.

## Results

### IL-27 induces STAT1-dependent expression of CIITA and IRF1 in IEC

The ability of IL-27 to induce CIITA and IRF1 expression in IEC [as seen in our previous microarray analysis (22)] was confirmed by qPCR and western blot in DLD-1 cells as well as in primary IEC (**Supplementary Figures S1A–F**).

**Figure 1:**
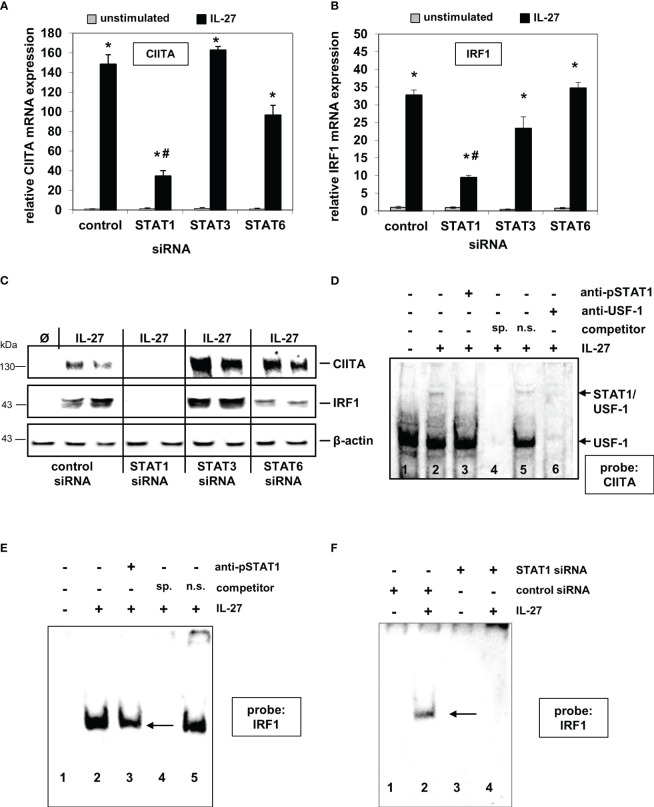
IL-27-induced CIITA and IRF1 expression is dependent on STAT1 activation and binding of STAT1 to the CIITA and IRF1 promoter. **(A, B)** Quantitative PCR of DLD-1 cells, transfected with siRNAs against STAT1, STAT3, or STAT6 prior to cytokine stimulation for 48 h, demonstrates that the presence of STAT1 is crucial for IL-27-induced CIITA **(A)** and IRF1 **(B)** mRNA expression. Gene expression was normalized to that in unstimulated control siRNA-transfected cells and is derived from three independent experiments. **(C)** Western Blot analysis of DLD-1 cells treated as in **(A/B)** shows that the induction of CIITA and IRF1 protein through IL-27 is abolished by a lack of STAT1 protein. Blot is representative of three independent experiments. **(D)** EMSA analysis shows that STAT1, together with USF-1, binds to the CIITA promoter upon IL-27 stimulation (lane 2). Addition of a STAT1 antibody (lane 3) or an excess of unlabeled specific competitor probe (lane 4) abolished the detection of STAT1. **(E)** EMSA analysis of the IRF1 promoter demonstrates binding of STAT1 to the IRF1 probe which could be reduced by a STAT1 antibody (lane 3) or a specific competitor probe (lane 4). **(F)** EMSA analysis of siRNA-transfected cells shows binding of STAT1 in IL-27-stimulated control cells while no protein binding to the probe was detected in STAT1 siRNA-transfected DLD-1 cells. All EMSAs are representative of three independent experiments Ø = unstimulated; sp.=specific; n.s.=non-specific *p<0.05 vs unstimulated # p<0.05 vs control+IL-27.

We previously demonstrated that IL-27 induces STAT1, STAT3, and STAT6 phosphorylation in IEC, thereby mediating differential gene expression (22). We therefore transfected DLD-1 cells with the respective STAT siRNAs prior to cytokine stimulation. IL-27-induced CIITA mRNA expression was strongly impaired in STAT1 siRNA-transfected cells in comparison to control siRNA-transfected cells (**Figure 1A**). Silencing of STAT3 or STAT6 had no significant effect on CIITA expression. Similar results were observed regarding IRF1 mRNA expression (**Figure 1B**). Western blot analysis confirmed a clear lack of IL-27-induced CIITA and IRF1 protein expression in STAT1 siRNA-transfected cells but not in those transfected with STAT3 or STAT6 siRNA (**Figure 1C**).

EMSA analysis with biotinylated probes (for sequences, see **Supplementary Table 1**) from the respective promoter sequences showed that IL-27-activated STAT1 binds directly to the CIITA and IRF1 promoter, respectively (**Figures 1D–F**). The CIITA probe revealed two protein complexes (**Figure 1D**), from which only the upper contained STAT1 and is induced by IL-27, while USF-1 is part of both protein complexes [as it has been described similarly for IFN-γ-induced activation of the CIITA promoter (30)]. EMSA with nuclear protein from cells in which STAT1 expression was silenced by siRNA transfection showed a complete lack of protein binding to the IRF1 probe (**Figure 1F**), further confirming that IL-27-activated STAT1 binds to the IRF1 promoter.

### IL-27 induces expression of MHC class II receptors and invariant chain (li) in IEC

CIITA is a known transactivator indispensable for HLA class II gene expression (12). We therefore determined mRNA expression of the HLA class II genes encoding for the alpha and beta chains of HLA-DR, -DP-, -DQ-, -DM, and -DO in IEC following IL-27 stimulation. Additionally, CD74, encoding the invariant chain involved in the formation and transport of MHC class II peptide complexes (31), was analyzed.

**Figure 2:**
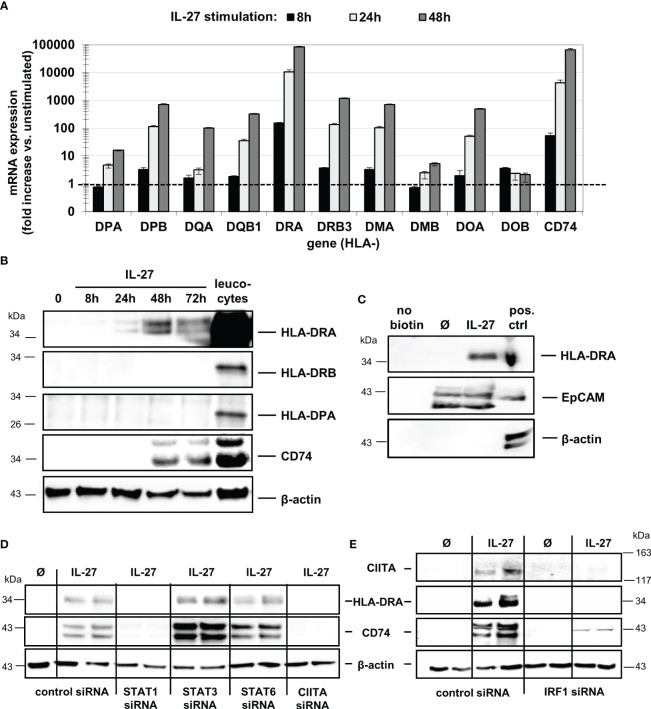
IL-27 induces expression of MHC class II mRNA and protein in DLD-1 cells, dependent on STAT1, CIITA, and IRF1. **(A)** Stimulation of DLD-1 cells with IL-27 for different time intervals reveals upregulation of HLA class II genes as determined by qPCR. Expression in unstimulated cells was set to 1.0 for each gene and is indicated by the dashed line. Data are presented as mean ± SEM from two independent experiment, each stimulation performed in triplicates, qPCR in duplicates per sample **(B)** Western Blot analysis with specific antibodies confirmed upregulation of HLA-DRA and CD74 proteins in IL-27-stimulated DLD-1 cells, while HLA-DRB and HLA-DPA proteins were not upregulated upon IL-27 stimulation for 48 h. Total protein isolated from leukocytes was used as positive control. **(C)** Cell surface proteins were biotin-labeled and isolated as described in the Methods section. Western Blot analysis shows that HLA-DRA is expressed on the cell surface. An EpCAM antibody was used as a positive control for a protein expressed on the cell surface, while a beta-actin antibody was used as a control for intracellular expression. **(D)** DLD-1 cells were transfected with siRNA targeting STAT1, STAT3, STAT6, or CIITA prior to IL-27 stimulation for 48 h. Western Blot analysis of siRNA-transfected cells confirms the need for STAT1 and CIITA presence to induce HLA-DRA and CD74 expression. **(E)** In IRF1 siRNA-transfected cells, no induction of CIITA, HLA-DRA, and CD74 protein could be observed following IL-27 stimulation for 48 h. All blots are representative images of at least three independent experiments.

While IL-27 up-regulated all mRNAs in a time-dependent manner (**Figure 2A**), only HLA-DRA and CD74 proteins were markedly expressed in IEC upon IL-27 treatment (**Figure 2B**), while other HLA receptors such as HLA-DRB and HLA-DP (**Figure 2B**) were not detected. The induction of HLA-DRA and CD74 gene expression by IL-27 was comparable to that of IFN-γ, a known inducer of MHCII gene expression (**Supplementary Figures S2A, B**). Moreover, we observed a synergistic effect of a combined treatment with IL-27 and TNF-α regarding CD74 protein induction (**Supplementary Figure S2C**). Several genes encoding costimulatory molecules (PD-L1, PD-L2, CD80, CD40, ICOSL, ICAM1) were upregulated through IL-27 stimulation on mRNA level (**Supplementary Figure S3A**). The ligand ICAM-1, which has been demonstrated to be involved in antigen presentation to T cells (32), was strongly upregulated on protein level (**Supplementary Figure S3B**)

Western Blot analysis with biotin-labeled cell surface proteins (**Figure 2C**) confirmed that HLA-DRA protein is transported to the cell surface, an indispensable prerequisite for antigen presentation and recognition by immune cells. The induction of HLA-DRA and CD74 protein expression was also confirmed by immunofluorescence in DLD-1 cells stimulated with IL-27 for 48 hours (**Supplementary Figures S4A, B**).

### IL-27-induced CIITA and MHCII expression is dependent on STAT1 and IRF1

To further analyze the role of the STAT1-CIITA pathway with regard to IL-27-induced HLA class II gene upregulation, we transfected DLD-1 cells with siRNA against STAT1, 3, 6 or CIITA prior to IL-27 stimulation for 48 hours. Silencing of CIITA as well as of STAT1 resulted in significantly decreased CD74 and HLA-DRA mRNA and protein expression (**Supplementary Figure S4C**, **Figure 2D**) following IL-27 stimulation, demonstrating that presence of STAT1 and CIITA is an important prerequisite for IL-27-mediated MHCII gene expression. Interestingly, an IRF1 binding site in the CIITA promoter has been reported (30), suggesting a crosstalk between HLAI and HLAII expression pathways. In IRF1-siRNA transfected cells, we observed significantly decreased mRNA and protein expression of CIITA and in consequence decreased CD74 and HLA-DRA expression levels (**Supplementary Figure S4C**, **Figure 2E**) upon IL-27 stimulation.

### Patients with active Crohn’s disease exhibit significantly higher CIITA, CD74, and HLA-DRA levels in intestinal IEC compared to controls

**Figure 3:**
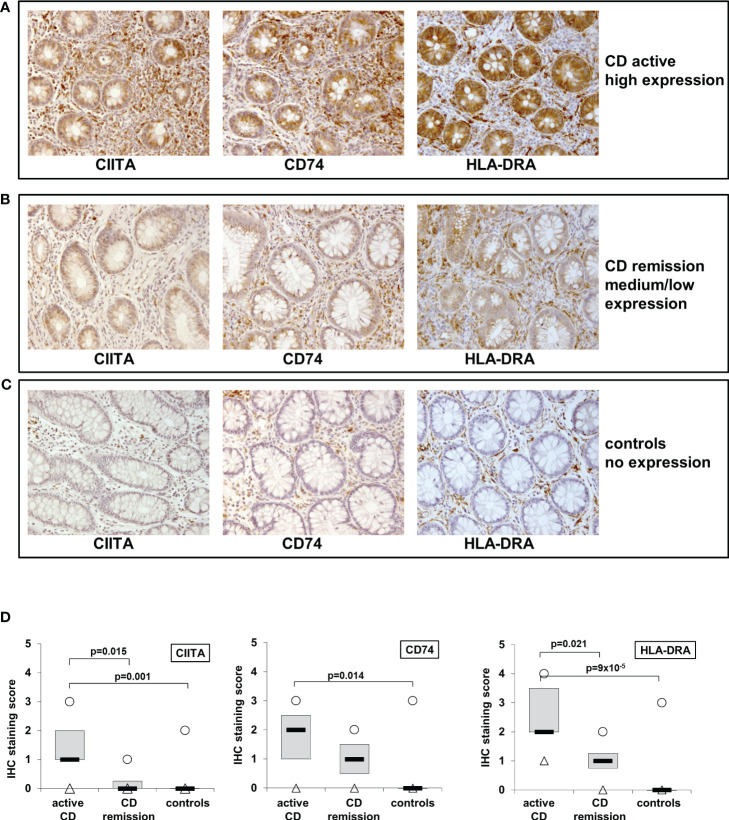
Immunohistochemistry of CIITA, CD74, and HLA-DRA expression in biopsies from patients with active and inactive Crohn’s disease (CD) and controls shows higher expression in active inflammation. **(A)** Representative images of high CIITA, CD74, and HLA-DRA expression in IEC in a patient with active CD. **(B)** Representative images of medium to low CIITA, CD74, and HLA-DRA expression in IEC in a CD patient in remission **(C)** Representative images of absent CIITA, CD74, and HLA-DRA expression in IEC in a biopsy from a healthy control. **(D)** Box plot of CIITA, CD74, and HLA-DRA expression in all analyzed biopsies (see **Table 1** and table legend for details). Median = black lines; first to third quartile = grey box; minimum = triangles; maximum = circles.

Previously, we had shown that IL-27 levels in intestinal biopsies from inflamed tissue of CD patients are significantly increased compared to non-inflamed tissue (22). As we here identified genes of the MHCII pathway as IL-27 target genes, we next analyzed the expression of the three IL-27 target genes CIITA, CD74, and HLA-DRA in IEC from CD patients. Biopsies from patients with active CD (n=11) or CD in remission (n=4) as well as from control patients with no history of IBD (n=10) were analyzed by immunohistochemistry. A staining score ranging from 0 (no staining) to 4 (very strong staining) was assigned to all samples with regard to staining intensity of IEC in the respective samples (**Table 1**). Representative images of high, medium/low, and no expression of the respective proteins are presented in **Figures 3A–C**. In summary, the average expression of all three proteins in IEC was significantly higher in patients with active CD compared to controls (**Figure 3D**, **Table 1**). CIITA and HLA-DRA levels in IEC were also significantly higher in patients with active CD compared to CD in remission (**Figure 3D**).

**Table 1:** Analysis of immunohistochemistry of human biopsies sampled from patients with active CD, CD in remission, and control patients presenting for routine endoscopic examination.

patient group	patient number	CIITA expression score	CD74 expression score	HLA-DRA expression score
**CD active**	**1**	2	3	4
	**2**	2	3	4
	**3**	1	0	1
	**4**	1	2	3
	**5**	2	3	2
	**6**	1	2	2
	**7**	1	1	2
	**8**	1	1	2
	**9**	1	1	3
	**10**	0	0	1
	**11**	2	2	4
	**mean ± SEM**	**1.27 ± 0.19**	**1.64 ± 0.34**	**2.55 ± 0.34**
**CD remission**	**1**	1	2	2
	**2**	0	n/a	1
	**3**	0	1	0
	**4**	0	0	1
	**mean ± SEM**	**0.25 ± 0.25**	**1.00 ± 0.50**	**1.00 ± 0.41**
**controls**	**1**	0	0	0
	**2**	0	0	0
	**3**	0	0	0
	**4**	0	0	0
	**5**	2	3	3
	**6**	0	0	0
	**7**	0	0	0
	**8**	0	0	0
	**9**	0	1	0
	**10**	0	0	0
	**mean ± SEM**	**0.20 ± 0.20**	**0.40 ± 0.31**	**0.30 ± 0.30**

Biopsies were stained with antibodies against CIITA, CD74, and HLA-DRA. The staining score defines the staining intensity of IEC in the respective samples. The score was determined by a researcher blinded to diagnosis and antibody and was set as follows: 0 = no staining; 1 = weak/partial staining; 2 = medium staining; 3 = strong staining; 4 = very strong staining; SEM, standard error of the mean

### IL-27 induces expression of MHC class I molecules in IEC dependent on IRF1 but independent of CIITA

Given that IRF1 is a major transcription factor involved in MHC class I gene expression (33) and it was induced by IL-27 in our experiments, we next analyzed the expression of the classical HLA-I receptors HLA-A,-B, C the nonclassical receptors HLA-E and HLA-F as well as of TAP1, TAP2, and B2M which are all involved in MHCI antigen presentation.

**Figure 4:**
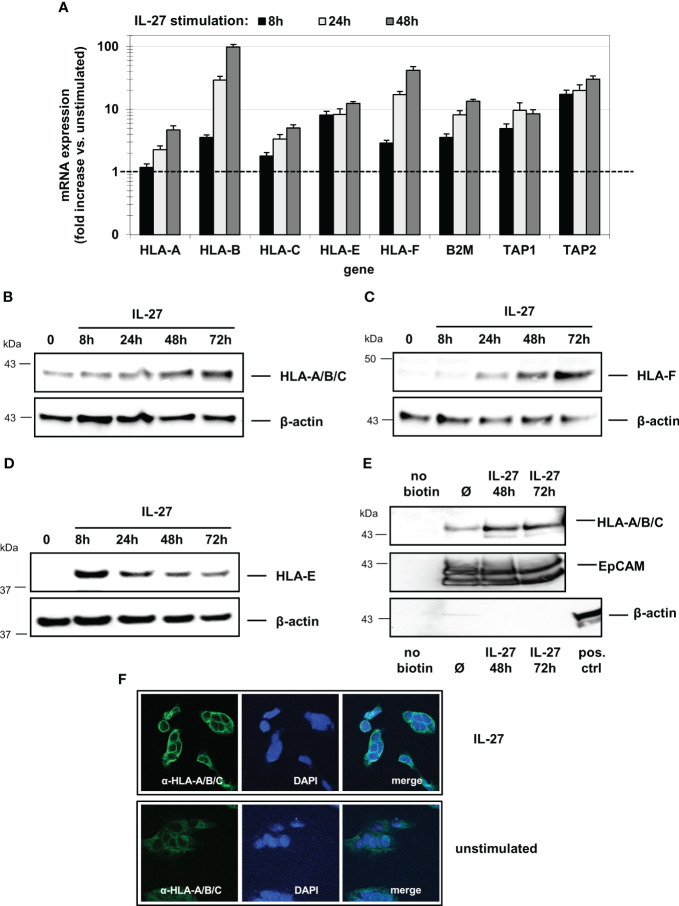
IL-27 induces the expression of HLA class I molecules in IEC. **(A)** DLD-1 cells were stimulated with IL-27 for the indicated time intervals. qPCR demonstrates upregulation of all HLA class I subtypes as well as of B2M, TAP1, and TAP2. Expression in unstimulated cells was set to 1.0 for each gene and is indicated by the dashed line. Data represent the mean ± SEM of three independent experiments with each three biological and two technical replicates **(B–D)** Total protein from IL-27-stimulated cells was isolated and analyzed by western blot, showing upregulation of **(B)** HLA-A/B/C, **(C)** HLA-F and **(D)** HLA-E protein. **(E)** Cell surface-expressed proteins were labelled with biotin and isolated as described in the methods section. Western blot analysis reveals expression of HLA-A/B/C on the cell surface. EpCAM was used as positive control for cell surface expression, while beta-actin is expressed only intracellularly. All western blots are representative of at least three independent experiments. **(F)** Immunofluorescence staining of DLD-1 cells shows higher expression of HLA-A/B/C protein in IL-27-stimulated cells compared to unstimulated cells. Ø = unstimulated.

All analyzed mRNAs were detected at basal levels in unstimulated DLD-1 cells and were induced in a time-dependent manner upon IL-27 stimulation (**Figure 4A**). Western Blot analysis revealed that HLA-A/B/C and HLA-F were maximally induced after 72 hours (**Figures 4B, C**), while HLA-E showed the highest protein levels after 8 hours with decreasing levels thereafter (**Figure 4D**). Analysis of biotin-labeled cell surface proteins revealed expression of the HLA-A/B/C receptors on the cell surface (**Figure 4E**). Immunofluorescence confirmed HLA-A/B/C induction in IL-27-stimulated cells (**Figure 4F**).

**Figure 5:**
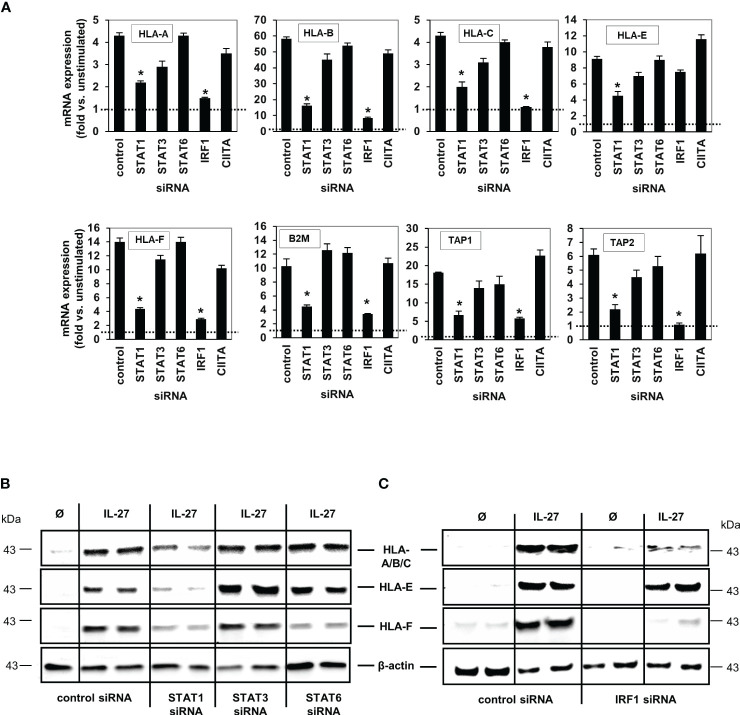
IL-27-induced MHC class I mRNA and protein expression is mediated via the STAT1 and IRF pathway. **(A)** DLD-1 cells were transfected with siRNA against STAT1, STAT3, STAT6, IRF1 or CIITA prior to IL-27 stimulation for 48 h. qPCR experiments demonstrate that the presence of STAT1 is crucial for all IL-27-induced HLA class I genes, while IRF1 is crucial for all except HLA-E. mRNA expression in unstimulated control cells was set to 1.0 for each gene and is represented by the dotted lines. * p<0.05 vs. control+IL-27. Data are presented as mean ± SEM from three independent experiments with three biological and two technical replicates **(B)** Western blot analysis confirmed that silencing of STAT1 inhibits IL-27-induced HLA-A/B/C, HLA-E and HLA-F protein expression. **(C)** The presence of IRF1 is necessary for IL-27-induced protein expression of HLA-A/B/C and HLA-F but not HLA-E as demonstrated by western blot. All blots are representative of at least three independent experiments with two biological replicates. Cells were stimulated with IL-27 for 48 h. Ø = unstimulated.

We then transfected DLD-1 cells with siRNA targeting STAT1, STAT3, STAT6, IRF1 or CIITA genes. Quantitative PCR (**Figure 5A**) and western blot analyses (**Figures 5B, C**) demonstrated that the IL-27-induced mRNA and protein expression of the HLA I receptors HLA-A, B, C, F as well as TAP1, TAP2, and B2M was dependent on the presence of STAT1 and IRF1, but was independent of CIITA. HLA-E was the only exception as its mRNA and protein expression was not dependent on the presence of IRF1 (**Figures 5A, C**)

### IEC are capable of antigen uptake and antigen processing

**Figure 6:**
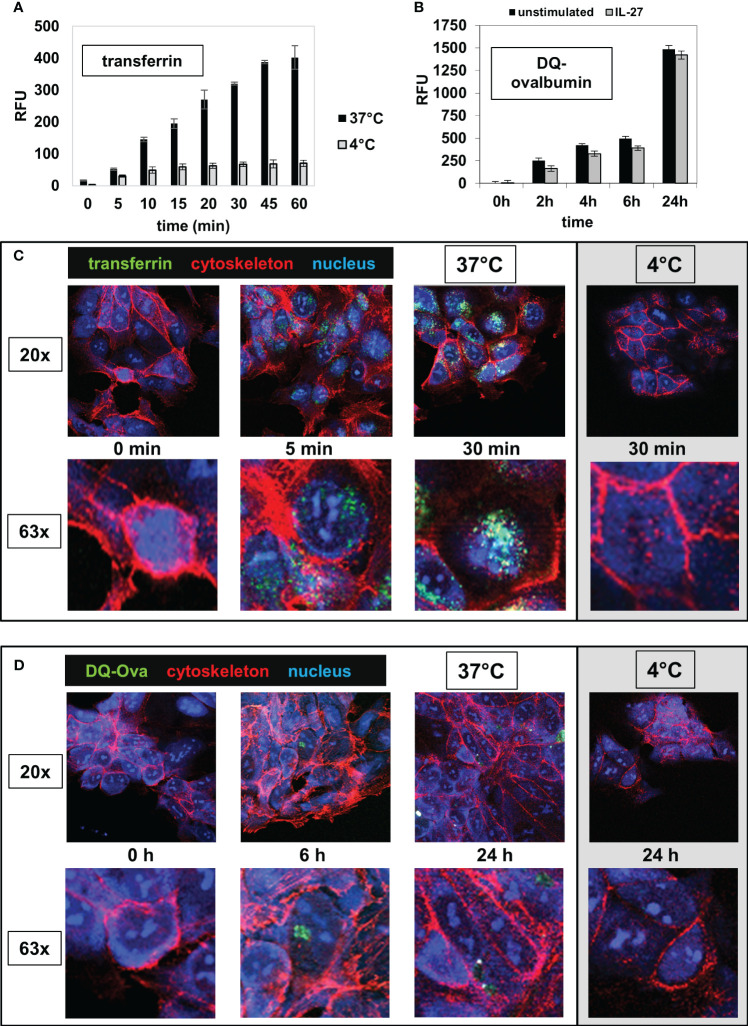
IEC are able to take up and to process extracellular antigens. **(A)** DLD-1 cells were incubated in 96 well plates with the AlexaFluor 488-labelled antigen transferrin (Tf) for time intervals as indicated at 37°C (black bars). The intracellular fluorescence (RFU) was measured in a microplate reader. Control reactions were performed at 4°C (grey bars). **(B)** DLD-1 cells (unstimulated or stimulated with IL-27 for 48 h) were incubated in 96 well plates with a self-quenched fluorescent conjugate of ovalbumin (DQ-Ova) that exhibits fluorescence only upon proteolytic degradation, for the indicated time intervals. The intracellular fluorescence was determined with a microplate reader. **(C)** DLD-1 cells were incubated on microscope slides with AlexaFluor-488-labelled transferrin (green) for time intervals as indicated and were fixed afterwards. The cytoskeleton was stained with AlexaFluor-546-labelled phalloidin (red) and the nucleus was stained with DAPI (blue). Control reactions were performed at 4°C. The upper panels show a 20x magnification and the lower panels show a detail (63x magnification) from the respective image above. **(D)** DLD-1 cells were incubated on microscope slides with AlexaFluor-488-labelled DQ-Ova for time intervals as indicated and were stained and analyzed as in **(C)**.

Next, we analyzed whether IEC are able to take up extracellular antigens from the surrounding environment, an important prerequisite for MHC-mediated antigen presentation. DLD-1 cells were incubated with the AlexaFluor 488-labeled antigens transferrin (Tf), dextran (Dx) or ovalbumin (Ova) for different time intervals at 37°C. A very rapid and extensive uptake of transferrin was observed after 5 minutes (**Figure 6A**, black bars). The uptake of ovalbumin (**Supplementary Figure S5A**) and dextran (**Supplementary Figure S5B**) was lower and had a slower kinetic. Cells in a control plate, which were treated identically but were incubated at 4°C instead of 37°C, displayed only very little uptake of extracellular antigen (**Figures 6A**, **S5A, B**, grey bars). To analyze whether IEC are capable of processing antigens following uptake, we incubated DLD-1 cells with the antigen DQ-Ova, a self-quenched fluorescent conjugate of ovalbumin that exhibits fluorescence only upon proteolytic degradation, i.e. antigen processing. An increase in fluorescence (indicating processing of DQ-Ova) was detected at low levels beginning after 2 hours and increased continuously during the total incubation time of 24 hours (**Figure 6B**). Pretreatment of the cells with IL-27 48 hours before the addition of antigen had no significant influence on antigen processing (**Figure 6B**). The results of the microplate reader were confirmed by imaging cells seeded on microscope slides and incubated with transferrin or DQ-Ova as described above (**Figures 6C, D**).

### IEC phagocytose apoptotic, necrotic, and necroptotic cells

**Figure 7:**
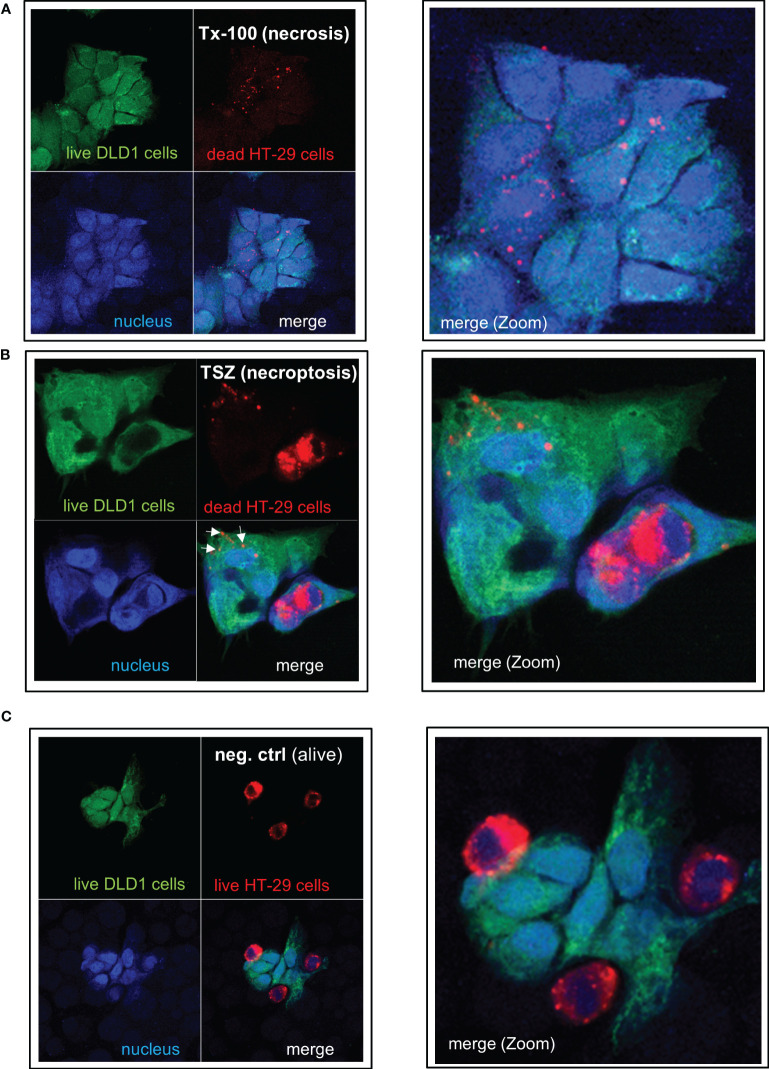
IEC are able to phagocytose apoptotic, necrotic or necroptotic cells. Alive DLD-1 cells (stained in green) were incubated with dead HT-29 cells (stained in red) for 24h at a ratio of 1:4. Cells were fixed and the nucleus was stained with DAPI. Imaging was performed by confocal LSM: The right panel represents a magnification of the left picture. **(A)** Necrotic cell death was induced in HT-29 cells by treatment with Triton X-100. **(B)** Necroptotic cell death was induced in HT-29 cells by simultaneous treatment with TNF-α, the Smac mimetic SM-164, and the caspase inhibitor z-VAD-fmk. **(C)** As a negative control, alive HT-29 (red) were cocultured with alive DLD-1 cells (green) demonstrating no uptake of cells or cell fragments. Tx-100: Triton X-100; TSZ: TNF-α+Smac-mimetic SM-164+caspase inhibitor Z-VAD-fmk.

To analyze whether IEC are (in addition to their capacity to take up and process small antigens) also able to phagocytose dead cell fragments or whole cells, we treated human colonic HT-29 cells (labeled with the red fluorescent dye PKH26) with TNF-α, the Smac mimetic SM-164, and, if applicable, the caspase inhibitor z-VAD-fmk to induce apoptotic or necroptotic cell death, respectively, as described **(** 26 **).** Nuclear morphology of the apopotic cells showed condensed chromatin, cell shrinkage, nuclear condensation, apoptotic body formation, and/or margination of nuclear chromatin as typical indicators of apoptotic cell death (**Supplementary Figure S6A**). Treatment with Triton X-100, freeze-thaw or heat was used to induce necrosis. Dead HT-29 cells were added to living green-labeled DLD-1 cells at a ratio of 4:1 and were incubated together for 24 hours. DLD1 cells ingested fragments of necrotic (**Figure 7A**) or necroptotic (**Figure 7B**, arrows) HT-29 cells as well as whole necroptotic (**Figure 7B**) or apoptotic cells (**Supplementary Figure S6B**). Living cells were not ingested (**Figure 7C**). Similar results were obtained when alive DLD-1 cells were incubated with dead DLD-1 cells (**Supplementary Figures S6C, D**). Prestimulation with IL-27 did not influence the effectiveness of phagocytosis (**Supplementary Figure S6E**).

### IL-27 enhances antigen processing via the immunoproteasome

We next determined the influence of IL-27 stimulation on the expression and the activity of the immunoproteasome (IP) in IEC, as antigenic peptides that are presented by MHC I receptors are usually generated by immunoproteasomal protein digestion (4).

**Figure 8:**
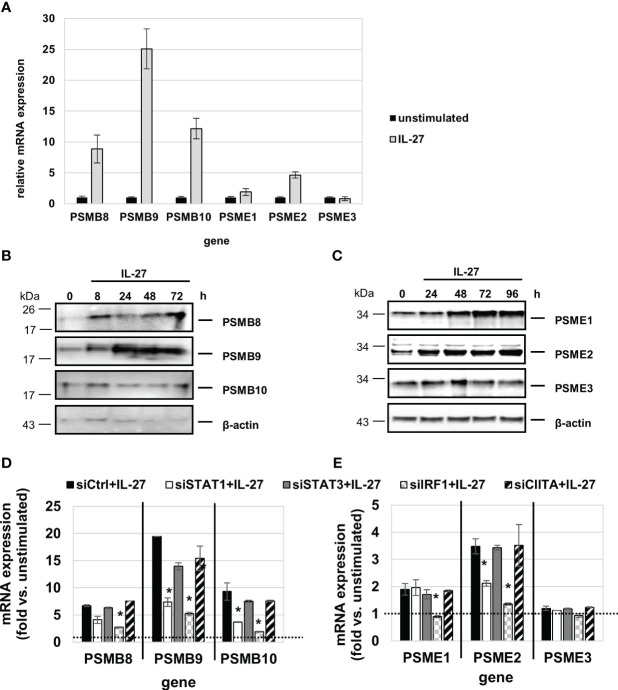
IL-27 induces the expression of immunoproteasome (IP) subunits in IEC via STAT1 and IRF1 signaling. **(A)** qPCR analysis of IL-27 stimulated DLD-1 cells reveals increased expression of all analyzed IP subunits with the exception of PSME3 after 48 h. **(B, C)** Protein from IL-27-stimulated cells was isolated and western blot analysis was performed. IL-27-induced protein expression of the IP core subunits PSMB8 and PSMB9 **(B)** as well as expression of the 19S regulator complex subunits PSME1 and PSME2 **(C)**. **(D, E)** DLD-1 cells were transfected with siRNA against STAT1, STAT3, IRF1 or CIITA or an unspecific control siRNA prior to IL-27 stimulation for 48 h. qPCR analysis demonstrates that mainly IRF1 (and to some extent STAT1) is indispensable for IL-27-induced IP subunit upregulation. Expression in unstimulated cell was set to 1.0 and is indicated by the dashed lines. * p<0.05 vs. control+IL-27.

QPCR analysis revealed that IL-27 induces mRNA expression of all three IP core subunits (β5i/PSMB8, β1i/PSMB9, β2i/PSMB10) in a time-dependent manner (**Figure 8A**). Moreover, the subunits PSME1 and PSME2 (but not PSME3) from the PA28 proteasome activator complex are induced by IL-27 (**Figure 8A**). In western blot experiments, an increase of PSMB8, PSMB9, PSME1, and PSME2 protein levels was observed upon IL-27 stimulation (**Figures 8B, C**). Transfection with siRNAs targeting STAT1, STAT3, IRF1 or CIITA revealed that the IL-27-induced expression of PSMB and PSME genes is dependent on IRF1 and in part on STAT1 (**Figures 8D, E**). The expression of the β5c, β1c, and β2c subunits of the constitutive proteasome was not influenced by IL-27 (**Supplementary Figure S7A**).

**Figure 9:**
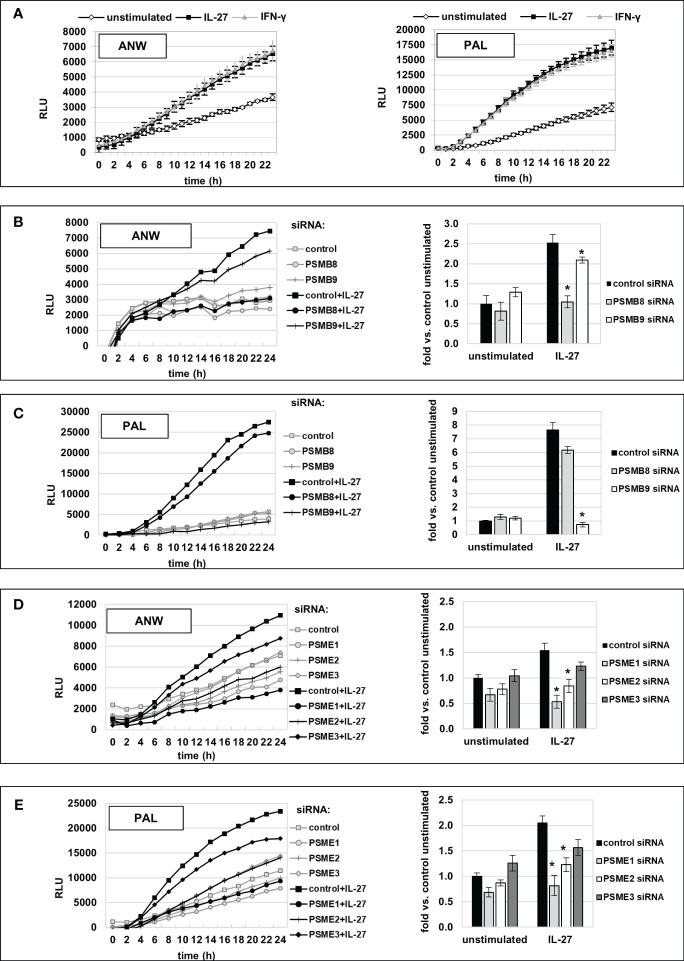
Increased immunoproteasome activity, induced by IL-27 stimulation in IEC, is mediated by PSMB8, PSMB9, PSME1, and PSME2 IP subunits. **(A)** Extracts of DLD-1 cells treated with IL-27 or unstimulated were incubated with the quenched fluorescent amino acid substrates alanine-asparagine-tryptophan (ANW, left panel) or proline-alanine-leucine (PAL, right panel), and fluorescence (indicating substrate processing) was measured in real-time on a microplate reader. IFN-γ was used as a positive control. IL-27 increased digestion of both substrates comparable to IFN-γ. **(B, C)** DLD-1 cells were transfected with siRNA targeting the IP core subunits PSMB8 or PSMB9, respectively, prior to IL-27 stimulation. IP activity regarding processing of the substrates (Ac-ANW)2R110 **(B)** and (Ac-PAL)2R110 was determined as in **(A)**. The left panels show the real-time fluorescence data, while the right panel represents the relative increase in fluorescence at the timepoint t=24h, compared to the unstimulated control. **(D, E)** DLD-1 cells were transfected with siRNA targeting the IP regulator subunits PSME1, PSME2, or PSME3, respectively, prior to IL-27 stimulation. IP activity, i.e. processing of the substrates (Ac-ANW)2R110 **(D)** and (Ac-PAL)2R110 **(E)**, was determined and is presented as in **(B)** and **(C)** Data are representative from one out of three experiments, each performed in biological and technical triplicates. * p<0.05 vs. control+IL-27.

We next measured IP activity using short peptides with amino acid sequences specific for the chymotrypsin-like (cleavage after hydrophobic amino acids, amino acid sequence ANW) and the branched-chain preferring cleavage activity (amino acid sequence PAL) of the immunoproteasome. Those peptides were coupled to the fluorophore R110 whose fluorescence is quenched but can be detected when the substrate is cleaved by IP activity. IL-27 stimulation resulted in a significant higher cleavage activity, similar to IFN-γ, for both substrates when compared to unstimulated cells (**Figure 9A**). Knockdown of IP subunit gene expression by specific siRNAs against PSMB8 or PSMB9 resulted in a significant decrease in the chymotrypsin-like activity for PSMB8 siRNA (**Figure 9B**), and PSMB9 siRNA transfection decreased the branched-chain amino acid preferring activity (**Figure 9C**) to nearly baseline. Knockdown of PSME1, 2 or 3 expression revealed that mainly PSME1 and PSME2 but not PSME3 contribute to IP substrate cleaving including both the chymotrypsin-like and branched chain amino acid-preferring activity of the immunoproteasome (**Figures 9D, E**). Silencing of IRF1 expression by siRNA had a strong negative effect on IP substrate cleaving (**Supplementary Figure S7**). Silencing of STAT1 or STAT3 had only minor effects, while silencing of CIITA had no impact on immunoproteasome activity (**Supplementary Figures S7B, C**).

### Antigen- and IL-27-primed IEC stimulate PBMC and CD4^+^ T cell proliferation

**Figure 10:**
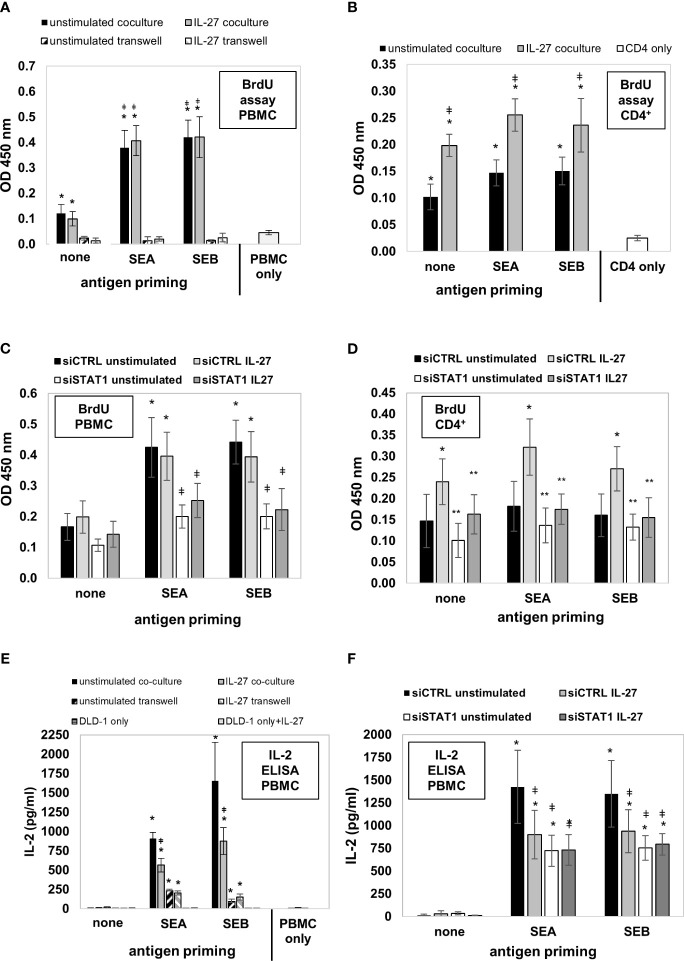
DLD-1 cells stimulate proliferation and IL-2 production of PMBCs or CD4^+^ T cells which is further increased by Staphylococcus antigen A or B (SEA, SEB) priming. **(A)** PBMC proliferation was determined by measuring BrdU incorporation. In direct coculture (filled bars), a higher proliferation of PMBCs was observed compared to PMBCs only. Priming of DLD1 cells with SEA and SEB further increased PBMC proliferation, while priming with IL-27 did not further influence PMBC proliferation. In indirect coculture (hatched bars), no proliferation above background was detected. * p<0.05 vs. PMBC only or transwell coculture; ǂ p<0.05 vs. no antigen-primed direct coculture. **(B)** CD4^+^ T cell proliferation was determined as in **(A)**. In direct coculture, a higher proliferation of CD4^+^ cells was observed compared to CD4^+^ cells only. Priming of DLD1 cells with SEA and SEB slightly increased CD4^+^ cell proliferation. Priming with IL-27 significantly increased CD4^+^ cell proliferation rate * p<0.05 vs. CD4^+^ cells only; ǂ p<0.05 IL-27 vs. unstimulated. **(C)** Transfection of DLD-1 cells prior to antigen priming significantly decreased PMBC proliferation rate. * p<0.05 vs no antigen; ǂ p<0.05 vs siCTRL. **(D)** Transfection of DLD-1 cells prior to antigen priming significantly decreased the CD4^+^ T cell proliferation rate. * p<0.05 vs unstimulated; ** p<0.05 vs siCTRL+IL-27 **(E)** IL-2 levels in the cell culture supernatants from the above described PBMC coculture experiments were highest in antigen-primed direct coculture (filled bars). IL-27 pretreatment significantly reduced IL-2 concentration (filled grey bars). In indirect coculture (hatched bars), IL-2 was also induced at lower levels by IEC antigen priming, but was not influenced by IL-27. * p<0.05 vs. no antigen coculture; ǂ p<0.05 vs. unstimulated antigen-primed coculture **(F)** Silencing of STAT1 in DLD-1 cells prior to coculture inhibited the IL-27-mediated downregulation of PMBC IL-2 levels.* p<0.05 vs no antigen priming ǂ p<0.05 vs siCTRL unstimulated. siCTRL, control siRNA; siSTAT1, STAT1 siRNA.

We then aimed to analyze whether the uptake, processing, and presentation of antigens in IEC leads to altered T cell immune responses. DLD-1 cells were left unstimulated or were primed with IL-27 for 72 hours to induce MHC receptor expression. In parallel, they were primed with Staphylococcus enterotoxin A or B (SEA, SEB) for 72 hours to allow binding, uptake, and processing of the respective antigens. Primed cells were mixed with freshly isolated PMBC or CD4^+^ T cells and were cultivated in direct or indirect coculture (see **Supplementary Figure 8** for an overview of the experimental workflow). Analysis of PBMC proliferation by measuring BrdU incorporation revealed that unstimulated PMBC alone did not proliferate noticably *in vitro* (**Figure 10A**, light grey bar). In contrast, in coculture with direct contact with unprimed DLD-1 cells, a higher BrdU incorporation was observed (**Figure 10A**, black and grey filled bars). Additional priming of DLD-1 cells with SEA or SEB antigen significantly boostered PBMC proliferation compared to unprimed cells, while pretreatment with IL-27 had no further effect (**Figure 10A**). In indirect coculture without direct cell-cell contact of DLD-1 cells and PBMC, the proliferation of PMBC was near to baseline, independent of DLD-1 antigen priming (**Figure 10A**; hatched bars). In coculture experiments of DLD-1 and CD4^+^ T cells, pretreatment of DLD-1 cells with IL-27 significantly increased CD4^+^ T cell proliferation, which was slightly further increased by antigen priming (**Figure 10B**).

Next, we transfected DLD-1 cells with STAT1 siRNA and repeated the described coculture experiments. Silencing of STAT1 significantly lowered antigen-induced PBMC proliferation (**Figure 10C**). Moreover, the IL-27 priming-induced proliferative effect on CD4^+^ T cells was significantly inhibited (**Figure 10D**), confirming that the STAT1 pathway, which we demonstrated as modulator of MHC receptor expression in the experiments shown above, mediates the proliferation-stimulating effect of IL-27 on CD4^+^ T cells.

### IL-27 significantly reduces IL-2 production in direct coculture of antigen-primed IEC and PBMC

Since IL-2 is an important T cell growth factor and a marker of T cell activation (34), we measured the concentration of IL-2 in the different supernatants from the cocultures described above by ELISA. No IL-2 or only trace amounts were detected in PMBC or DLD-1 individual cultures, or in direct cocultures of PBMC together with unprimed DLD-1 cells (**Figure 10E**). Strongly increased levels of IL-2 were present in direct cocultures of antigen-primed DLD-1 cells and PBMC (**Figure 10E**, filled bars). Priming of DLD-1 cells with IL-27 (in addition to antigen priming) resulted in significantly decreased IL-2 levels compared to antigen-primed DLD-1 cells without IL-27 pretreatment in direct coculture (**Figure 10E**, black bars vs. grey bars). In indirect primed coculture, lower but still significantly increased levels of IL-2 were measured compared to individual cell culture or indirect coculture without antigen priming (**Figure 10E**, hatched bars). Silencing of STAT1 (the transcription factor necessary for MHC gene expression) in DLD1 cells prior to stimulation abolished the IL-2-lowering effect of IL-27, confirming an involvement of this signaling pathway (**Figure 10F**).

## Discussion

Colonic IEC do not express MHCII molecules under healthy, homeostatic conditions, while their expression is upregulated in human and in murine colonic IEC under inflammatory conditions [reviewed in (35)]. IFN-γ has been identified as a major regulator of inducible MHCII expression as well as an important regulator of MHCI expression (36). However, very little is known about other factors and pathways regulating intestinal epithelial MHCII expression.

In this study, we identified the cytokine IL-27 as a so far unknown inducer of MHCI and MHCII expression via the master transcription factors IRF1 and CIITA, respectively, in IEC lines and primary IEC. This is in line with studies from other human cell types such as endothelial cells (16), THP-1 monocytic cells (17), or keratinocytes (37), which have shown MHCI and/or MHCII upregulation upon IL-27 stimulation. However, in these studies, detailed pathway and functional analyses are largely missing. Mechanistically, we demonstrated that the upregulation of MHCI and MHCII in IEC by IL-27 is mediated via STAT1 that directly binds to the promoters of IRF1 and CIITA genes. Interestingly, while CIITA was necessary exclusively for MHCII expression, there was a crosstalk between the IRF1/MHCI and the MHCII pathway, as silencing of IRF1 also decreased IL-27-induced CIITA and MHCII expression. This is plausible as an IRF1 binding site in the CIITA promoter, which is necessary for IFN-γ-induced CIITA expression, has been described (30).

While most mRNAs encoding for the α and β chains of the different MHCII receptor subtypes as well as the invariant chain li (CD74) were strongly induced by IL-27 (most of them *de novo*), only HLA-DRA and CD74 were translated into protein, suggesting a posttranscriptional regulation. This is in line with other studies demonstrating a differential ability of IEC (and other adherent cell types) to express different HLA-D receptor subtypes *in vivo* and *in vitro*. As a rule, higher HLA-DR, and little or no HLA-DP and HLA-DQ receptor expression was found (7, 38).

CD74 acts as a chaperone and is involved in the formation and transport of MHCII peptide complexes (39). The strong IL-27-induced increase in CD74 expression was further enhanced by combined treatment with TNF-α, a cytokine that is very abundant in IBD patients. It has been recently demonstrated that CD74 expression in IEC is crucial for inflammation-induced mucosal healing in different murine colitis models (40), further supporting a protective role of IL-27 in IEC as previously demonstrated by us (22).

In colonic tissue from CD patients, we detected significantly increased expression of CIITA, HLA-DRA, and CD74 in IEC, compared to healthy controls. Moreover, expression of CIITA and HLA-DRA was significantly higher in patients with active disease, compared to CD patients in remission. These data correlate with results from our previous study demonstrating increased IL-27 expression in inflamed colonic tissue from CD patients as well as an increased IL-27 receptor expression in IEC (22). In line with our data, other groups also demonstrated increased MHCII and/or CD74 expression in inflamed IBD tissue (7, 41–43). Mouse models of intestinal inflammation, however, revealed contradictory results regarding the role of IEC MHCII expression. While one study demonstrated that an IEC-specific knockout of MHCII worsened colitis in T cell adoptive transfer model (44), another study reported an ameliorated T cell and DSS colitis in IEC-specific MHCII knockouts (45).

In contrast to MHCII, we detected all classes of MHCI receptors in unstimulated IEC on protein level, and their expression was further upregulated through IL-27 stimulation. While this was dependent on the STAT1/IRF1 axis for HLA-A, -B, -C, and -F, the expression of HLA-E was independent of IRF1 and differed in the kinetics, compared to the other MHCI genes. This is in line with previous data showing that STAT1 but not IRF1 binds HLA-E promoter upon IFN-γ stimulation (46). HLA-E belongs to the group of non-classical MHCI molecules and specifically binds peptides derived from the leader sequences of other MHCI molecules (47), thereby representing a central innate mechanism for monitoring MHCI expression within a cell. The peptide-receptor complex is recognized by NK cells via the CD94-NKG2 receptor (48). Upregulation of HLA-E results in an inhibition of NK cell-mediated lysis (49). Interestingly, it has been demonstrated that IEC derived from patients with UC fail to express HLA-E, in contrast to IEC from controls or CD patients (50), suggesting a protective role for this receptor in the pathogenesis of UC. In agreement with these results, we have demonstrated that HLA-E-inducing IL-27 levels are increased in inflamed colonic biopsies from CD, but not in UC patients (22).

As an important prerequisite for the presentation of exogenous antigens, we show here that IEC are able to ingest and to efficiently process extracellular soluble antigens, as well as to phagocytose necrotic, necroptotic, or apoptotic cells. The importance of IEC to act as phagocytic cells in the context of intestinal inflammation has been demonstrated in a murine DSS colitis model (51). Inhibition of IEC phagocytosis by genetic ablation of the phagocytic receptor BAI1 in IEC resulted in worsened DSS colitis with many uncleared apoptotic corpses and inflammatory cytokines within the colonic epithelium (51). A forced overexpression of BAI1 resulted in fewer apoptotic cells, reduced inflammation, and attenuated disease (51).

While the rate of extracellular antigen uptake in IEC was not influenced by IL-27 treatment, we here identified the so far unknown capability of IL-27 to increase the rate of intracellular antigen processing via the immunoproteasome. The role of the immunoproteasome in intestinal inflammation has been analyzed in several studies. An increased expression of immunoproteasome subunits in the colon and terminal ileum was observed in inflamed tissue of CD but not UC patients (52). Experimental colitis in mice can be prevented or ameliorated by selective inhibitors of the immunoproteasome (53–56). In line with this, LMP7 (=PSMB8) knockout mice are protected from DSS colitis (57). However, the expression of LMP7 in hematopoietic cells (but not other cells such as IEC) was critical for colitis development (57), suggesting differential roles of the immunoproteasome in different cell types. Moreover, other major functions of immunoproteasomes such as the protection from oxidative stress and oxidation-induced cell death (58, 59) as well as a role in NF-κB activation (55, 60) have been described. Therefore, the functional consequence of IL-27-induced immunoproteasome upregulation and increased antigen processing in IEC needs to be further defined.

In coculture experiments of IEC (naive or antigen-primed) with PBMC or CD4^+^ T cell, we observed minor proliferative effects of unprimed DLD-1 on PBMC expansion. On the other hand, PBMC proliferation was strongly enhanced by prior antigen priming and additionally resulted in very high production of IL-2. However, while pretreatment of IEC with IL-27 did not influence PBMC proliferation (in contrast to increased CD4^+^ proliferation), the production of IL-2 was markedly reduced. This is in line with a study showing that IL-27 pretreatment of hepatocellular carcinoma cells resulted in lowered IL-2 production by anti-CD3/-CD28 activated T-lymphocytes (61). Our coculture experiments also demonstrated that a direct cell-cell contact of IEC and PBMC is necessary for IEC-stimulated PBMC proliferation, suggesting a receptor-mediated stimulation of PBMC or CD4^+^ T cell proliferation by DLD-1 cells that is enhanced through antigen priming. Similar observations have been reported for IFN-γ-treated keratinocytes that were able to activate T cells and to induce their proliferation upon priming with staphylococcal superantigens, depending on direct cell-cell contact (62, 63).

In contrast to PBMC coculture, IL-27 pretreatment of DLD-1 cells markedly increased CD4^+^ cell proliferation in coculture experiments which could be abolished by silencing of the STAT1 pathway. This differential effect of IL-27 on total PBMC and CD4^+^ cell proliferation might be explained by the presence of other MHC positive cells (such as professional APC) in the PBMC preparation which may mask the effect of IEC. This is also reflected by the overall higher BrdU incorporation of PBMC compared to CD4^+^ cells in IEC coculture.

Overall, the IL-27-induced gene expression pattern and the employed STAT1/IRF/CIITA signalling pathways identified here in IEC resemble that described for IFN-γ in different cell types such as hepatocytes or ovarian cancer cells (15, 61, 64, 65). In line with this, Hall et al. discovered that both IFN-γ and IL-27 promote a similar population of T-bet(+) CXCR3(+)Treg cells that limit T helper 1 (Th1) cell-mediated pathology (66). Very interestingly, the development of these Tregs was dependent on IFN-γ in the periphery but on IL-27 at local mucosal sites of inflammation (66). Another very recent study identified IEC as one of the major sources of IL-27 in gut-associated tissue (20), suggesting an important local role of this cytokine in intestinal mucosal immunity. This epithelial-derived IL-27 specifically promoted the differentiation of a distinct CD8αα+CD4+ intraepithelial lymphocyte (IEL) population that conferred intestinal barrier immunity and was indispensable for providing optimal immune response against enteric pathogens (20). Interestingly, IL-27 produced by conventional dendritic cells or myeloid cell was dispensable for this process. Another recent study reported that an IEC-specific deletion of MHCII (and PD-L1) hindered the development of those CD8αα+CD4+ IELs (67). One might speculate that, at least in part, IL-27-induced MHCII expression in IEC (as identified in this study) might be important for the development of this specific IEL subset. It will therefore be of great interest to analyze the functional consequences of an IEC-specific IL-27 receptor knockout in the context of intestinal immunity, with particular consideration of IEC MHC receptor expression.

Taken together, our data (summarized in **Supplementary Figure S9**) reveal a novel role for IL-27 in mediating intestinal epithelial antigen processing and presentation via MHCI and MHCII receptors, thereby underscoring the importance of IEC as non-professional APCs.

## Data availability statement

The original contributions presented in the study are included in the article/**Supplementary Material**. Further inquiries can be directed to the corresponding author.

## Ethics statement

The studies involving humans were approved by Ethics committee of the Ludwig-Maximilians-University Munich, Department of Medicine. The studies were conducted in accordance with the local legislation and institutional requirements. The human samples used in this study were acquired from primarily isolated as part of the ethics protocol of our previous study (22) for which ethical approval was obtained. Written informed consent for participation was not required from the participants or the participants’ legal guardians/next of kin in accordance with the national legislation and institutional requirements.

## Author contributions

JD conceived the study, planned and conducted experiments, analyzed the data, and wrote the manuscript. SB conceived the study, helped writing the manuscript and provided funding. All authors contributed to the article and approved the submitted version.
