# Supplementary material for: Identification of IL-27 as a novel regulator of major histocompatibility complex class I and class II expression, antigen presentation, and processing in intestinal epithelial cells
Source: Front Immunol. 2023 Sep 25;14:1226809. doi: 10.3389/fimmu.2023.1226809 (PMC10561092; doi:10.3389/fimmu.2023.1226809)

Supplementary Material

Identification of IL-27 as a novel regulator of major histocompatibility complex (MHC) class I and class II expression, antigen presentation and processing in intestinal epithelial cells

Julia Diegelmann*, Stephan Brand

*** Correspondence:** Julia Diegelmann: julia.diegelmann@med.uni-muenchen.de

1 Supplemental material and methods

Reagents and antibodies

Recombinant human IL-27, IFN-γ, and TNF-α were from R&D Systems (Wiesbaden, Germany). Small inhibitory (si) RNAs targeting STAT1, STAT3, STAT6, CIITA, IRF1, PSMB8, PSMB9, PSMB10, PSME1, PSME2, PSME3, and unspecific control siRNA were from Ambion (Applied Biosystems, Darmstadt, Germany). Lipofectamine RNAiMax was from Life Technologies/Invitrogen (Darmstadt, Germany).

Beta-actin and IRF1 antibodies were from Cell Signaling Technology (NEB, Frankfurt, Germany). Phospho-STAT1 antibody was from BD Biosciences (Heidelberg, Germany) while HLA-F antibody was from Proteintech Group (Manchester, U.K.). EpCam antibody was from Novus Bio (Biotechne, Wiesbaden, Germany). PSMB8, PSMB9 and PSMB10 antbodies were from Viva Biosciences (Exeter, U.K.). PSME1 and PSME3 antibodies were from Bethyl Laboratories (Montgomery, TX, U.S.A.) while PSME2 antibody was from Abgent (San Diego, CA, U.S.A.). All other primary antibodies used in this study (CIITA, USF-1, HLA-DQA1/2, HLA-DPA1, HLA-DRA, HLA-DRB, HLA-A/B/C, HLA-E, TAP1, TAP2, B2M, CD74) were purchased from Santa Cruz (Heidelberg, Germany).

Anti-mouse and anti-rabbit HRP-coupled secondary antibodies were from GE Healthcare (Freiburg, Germany) while anti-goat -HRP-coupled antibody was from Santa Cruz. Anti-mouse AlexaFluor-488 coupled antibody was from ThermoFisher Scientific (Bonn, Germany). Green fluorescent dye ViaFluor-488 SE was from Biotium (Fremont, CA, U.S.A.), red fluorescent PKH26 was from Sigma-Aldrich (Schnelldorf, Germany). Immunoproteasome substrates ((Ac-ANW)2R110, (Ac-KQL)2R110, (Ac-PAL)2R110) were from AAT Bioquest (Sunnyvale, CA, U.S.A.).AlexaFluor-488-labeled Transferrin (Tf), Dextran (Dx) and Ovalbumin (Ova) as well as BODIPY® FL dye-labeled DQ-Ovalbumin (DQ-Ova) were obtained from Molecular Probes/ThermoFisher Scientific. PCR primers and biotin-labeled probes for gelshift assays were synthesized by TIB Molbiol (Berlin, Germany).

Quantitative PCR

Real-time qPCR (for primer sequences see supplemental Table 1) was performed on a LightCycler480 instrument with SYBR Green PCR Master Mix from Roche using 3 µM of each forward and reverse primer. All primers were designed not to amplify genomic DNA, according to the published sequences. A standard curve was generated for each primer. Expression levels were normalized to the expression of the housekeeping genes β-actin and/or RPL13A in the respective samples. mRNA fold changes were calculated using the □□C_T method. When no basal expression was detected, a C_T-value of 45 cycles was used for calculation. P values were calculated with the unpaired, two-tailed Student's t test.

Nuclear extracts and electrophoretic mobility shift assay (EMSA)
Nuclear extracts were essentially isolated as described {Diegelmann, 2012 #1970}. Briefly, cells were incubated in hypotonic buffer for 15 minutes. NP-40 was added to disrupt the cell membrane. The nuclei were pelleted by centrifugation and were incubated for 15 minutes in high salt buffer on a rocking platform followed by centrifugation to extract the nuclear protein. Nuclear extracts were frozen at -80°C until further use. For EMSA, 15-20 µg of nuclear extract were incubated with biotinylated double stranded oligonucleotides (for sequences, see supplemental Table 1) and 1 µg of poly(dI-dC) in 1x binding buffer (10 mM Tris pH 7.4, 50 mM KCl, 1 mM DTT) for 30 minutes. In some experiments, 1 µg of phospho-STAT1 antibody, 1 µg of USF-1 antibody or a 50-fold excess of unlabeled competitor oligo was added 15 minutes prior to the addition of the labeled oligo. The samples were run on a precast 6% polyacrylamide gel (Invitrogen) in 0.5x TBE buffer and transferred to a positively charged nylon membrane. Biotin detection was performed with the LightShift Chemiluminescent EMSA kit from Pierce/ThermoFisher Scientific.

Biotin labeling of surface proteins
Proteins expressed on the cell surface were biotin-labeled with the Pierce Cell Surface Protein Biotinylation and Isolation Kit (ThermoFisher Scientific) following the manufacturer's guidelines. Briefly, adherent cells were incubated with Sulfo-NHS-SS-Biotin for 10 minutes at room temperature. After washing, cells were collected in centrifugation tubes and were lysed in lysis buffer. The cleared supernatant was applied to columns containing NeutrAvidin agarose and was incubated for 30 minutes at room temperature with end-over-end mixing on a rotator. After four washing steps, the bound protein was eluted with elution buffer for 30 minutes at room temperature with end-over-end mixing. The protein content was determined by the Bradford method.

Immunoproteasome (IP) activity assay
IP activity was measured using short, IP subunit-specific amino acid sequences coupled to a fluorophore whose fluorescence is quenched but can be detected when the substrate is cleaved by IP activity. For details, see supplemental data. DLD-1 cells in 6 well plates were stimulated with IL-27 (or were left unstimulated) for 48 hours and were subsequently lysed in 1x ReadiUse™ Mammalian Cell Lysis Buffer (AAT Bioquest) on ice for 20 min. The lysate was cleared by centrifugation and the supernatant was immediately used for the assay.
A 2x reaction mixture was prepared consisting of 25 µM fluorescent substrate, 4 mM EDTA and 10 mM DTT in 25 mM HEPES (pH 7.4). Equal volumes (50 µl) of the 2x reaction mixture and the cell lysate were mixed. Fluorescence development was measured in real-time every hour for a total of 24 hours in a microplate reader (Infinite 200, TECAN, Austria) with temperature set to 37°C and periodic shaking (excitation/emission wavelengths 498/520 nm). Results were normalized to the protein content in the respective samples.

Cell labeling with ViaFluor-488 SE and PKH26 fluorescent dyes
ViaFluor-488 SE is a dye that is initially non-fluorescent and diffuses passively into live cells where it is converted to fluorescent dye by intracellular esterases. The dye then covalently react with amine groups on proteins, forming fluorescent conjugates that are retained in the cell.
DLD-1 cells were seeded on microscope slides with a density of $2x10^4$/ml and were allowed to attach overnight. The next day, medium was removed and cells were incubated with 4 µM ViaFluor-488 SE in PBS for 15 minutes at room temperature. The staining solution was replaced with fresh, pre-warmed cell culture medium and slides were incubated for 5 minutes at 37°C to hydrolyze free dye. Again, cell culture medium was replaced by fresh medium and

cells were incubated for at least 30 minutes at 37°C to allow the dye to react with intracellular proteins.
HT-29 cells were stained with the red fluorescent dye PKH26 as follows: Cells were trypsinized, washed and $2x10^7$ cells were resuspended in 1 ml Diluent C (Sigma-Aldrich). They were added to 1 ml of prepared PKH26 dilution resulting in a final concentration of $2x10^6$ M PKH26. After 5 minutes, the staining reaction was stopped by adding an equal volume of serum. After several washes in complete medium, cells were assessed for viability and recovery.

Analysis of antigen uptake and processing

Transferrin (Tf), dextran (Dx) and ovalbumin (Ova) antigens were labeled with AlexaFluor-488 fluorescent dye. DQ-ovalbumin (DQ-Ova) was labeled with BODIPY® FL dye, a self-quenched conjugate of ovalbumin that exhibits bright green fluorescence upon proteolytic degradation, i.e. antigen processing. DLD-1 cells were incubated with the respective antigens at concentrations of 20 µg/ml (Ova, DQ-Ova) or 25 µg/ml (Tf, Dx) in 96 well plates or on microscope slides, respectively.
For the analysis of uptake kinetics, antigen incubation was performed between 2 minutes and 24 hours at 37°C. As a negative control, incubation was performed in the same way at 4°C. After the respective incubation times, cells in 96 well plates were washed with PBS and fluorescence was measured in a microplate reader (TECAN) with excitation and emission wavelengths of 490 and 525 nm, respectively. The background fluorescence of blank wells containing cells but no fluorophore was subtracted from the measurements.
Alternatively, cells on slides were washed with PBS, fixed in 4% paraformaldehyde for 15 minutes and permeabilized with 0.1% Triton X-100 in PBS for 5 minutes, followed by incubation with phalloidin (labeled with AlexaFluor-555 (Molecular Probes)) and DAPI to stain the cytoskeleton and the nucleus, respectively. Slides were mounted in ProLong Gold antifade mountant (Molecular Probes) and pictures were taken on a LSM510 confocal laser scanning microscope (Zeiss, Jena, Germany).

Apoptosis and necrosis induction and phagocytosis assay

To induce apoptotic/necroptotic/necrotic cell death, PKH26-labeled HT-29 cells were seeded in 6 well plates at a density of $4x10^5$/ml. Apoptotic cell death was induced by combined treatment with 20 ng/ml TNF-α (T) and 50 nM of the Smac mimetic SM-164 (S) as described {Zargarian, 2017 #895}. Necroptotic cell death was induced by combined treatment with 20 ng/ml TNF-α (T), 50 nM SM-164 (S), and 20 µM of the caspase inhibitor Z-VAD-fmk (Z) as described {Zargarian, 2017 #895} for 24 hours. As a positive control for cell death (necrosis), cells were treated with 0.01% Triton X-100 or were subjected to a freeze-thaw procedure. After 24 hours, floating (dead) cells in the cell culture medium were collected by centrifugation and counted. They were added to DLD-1 cells (earlier seeded on microscope slides and labeled with ViaFluor-488 SE) at a ratio of 4:1. After 24 hours of coculture, slides were washed with PBS, fixed with 4% paraformaldehyde and the nucleus was counterstained with ToPro3. Cells were analyzed by confocal laser scanning microscopy on a Zeiss LSM510 confocal laser scanning microscope.

Immunohistochemistry

Slides with paraffin-embedded biopsies from CD patients and healthy controls were deparaffinized in xylene and ethanol according to standard procedures. Heat-induced antigen retrieval was performed in citrate buffer pH 6.0 for 20 min. After washing in distilled water, endogenous peroxidases were blocked in 3% H_2O_2. After blocking in 10% rabbit serum in TBS/0.05% Tween (TBS-T) followed by an avidin-biotin block (15 min each), the slides were

incubated with the primary antibody overnight at 4°C. After several washes in TBS-T, a biotin-conjugated secondary antibody was added for 30 minutes at room temperature. After washing and incubation with streptavidin-HRP, signal was developed with the peroxidase substrate diaminobenzidine (DAB). Slides were counterstained with haematoxylin. Images were acquired on a Zeiss Axioskop 40 microscope.

Peripheral blood mononuclear cells (PBMC) and CD4$^+$ T cell isolation

PBMC and CD4$^+$ T cells were isolated by density gradient centrifugation using Lymphoprep density gradient medium (Stemcell Technologies, Vancouver, Canada) as follows: Leucosep tubes (containing a porous barrier that prevents mixture of the sample material with the separation medium (BD Biosciences) were filled with density gradient medium and were centrifuged briefly to collect the separation medium below the integrated porous barrier. For the isolation of PBMC, collected whole blood samples were mixed with an equal volume of PBS / 2 % FBS and were added on top of the porous barrier of the Leucosep tubes. For the enrichment of CD4$^+$ T cells, blood samples were mixed with RosetteSep CD4$^+$ T cell enrichment cocktail (Stemcell Technologies) and were incubated for 10 minutes at room temperature before mixing with an equal volume of PBS / 2% FBS and transferring to Leucosep tubes. Tubes were centrifuged at 1,200 *g* for 10 minutes. The enriched mononuclear cell layer was transferred to a new tube and was washed twice with PBS / 2% FBS. Finally, CD4$^+$ T cells or PBMC were resuspended in RPMI medium containing 10% FBS and 1% penicillin/streptomycin. PBMC were incubated for an additional 2-3 hours in a cell culture plate in a humidified 5% CO_2 incubator at 37°C to allow adherence of monocytes. The supernatant containing non-adherent PBMC was transferred to a new tube and counted.

Direct and indirect coculture of IEC and CD4$^+$ T cells or PBMC

DLD-1 cells were seeded in 12 well plates and were treated with or without 50 ng/ml IL-27. Simultaneously, in some wells, Staphylococcus enterotoxin A or B (Sigma Adrich) at a concentration of 30 ng/ml or tetanus toxoid (Calbiochem/ Merck, Darmstadt, Germany) at a concentration of 4 µg / ml was added to the cells. They were incubated for 72 hours to allow binding, uptake and processing of the respective antigens.
After the incubation period, 4 µg / ml mitomycin C was added to inhibit DNA synthesis and thereby to reduce BrdU incorporation into DLD-1 cells. After 2 hours, DLD-1 cells were washed extensively with PBS to remove remaining cytokine, antigens, and mitomycin C. Cells were harvested by trypsination and counted. For direct coculture experiments, they were seeded in 96 well plates at a density of 2 x 10^4 DLD-1 cells per well with 8 wells per treatment group. An equal volume of freshly isolated PBMC or CD4$^+$ T cells containing 1 x 10^5 cells was added to the pre-seeded DLD-1 cells to obtain a cell number ratio of 1:5. Alternatively, both cell types were seeded in 12 well plates at the same ratio (1.5x10^5 DLD-1 cells and 7.5x10^5 PBMC or CD4+T cells per well). Control wells contained either only DLD-1 cells or only PBMC / CD4$^+$ T cells. Wells with no cells served as blank controls. PBMC stimulated with concanavalin A served as positive control. Cells were cultured between 48 and 72 hours before analysis.
For indirect coculture, 12 well plates with Transwell inserts (0.4 µm pore size) were used. DLD-1 cells were seeded on the bottom of the wells, while PBMC or CD4$^+$ T cells were cultured in the insert to prevent direct cell-cell contact.

Analysis of PBMC and CD4$^+$ T cell proliferation in IEC coculture

Cell proliferation was determined with the colorimetric BrdU cell proferation ELISA (Roche), according to the manufacturer's recommendations. Briefly, BrdU labeling solution was added to the cells 16 hours before detection. The next day, the cell culture medium containing the suspension cells was transferred to a new 96 well plate. The plate was centrifuged at 300 *g* for 10 minutes to pellet the cells. The supernatant was flicked off and cells were dryed for 1 hour

at 65°C. Cells were fixed and were then incubated with a peroxidase-conjugated anti-BrdU antibody for 90 min. After several washes, detection was performed with TMB substrate. Reactions were stopped with 1 M H_2SO_4 and absorbance was read in an Varioscan ELISA reader (ThermoFisher) at 450 nm (reference wavelength 690 nm). The absorbance from wells containing only DLD-1 cells was subtracted from the absorbance from well containing DLD-1 plus PBMC / $CD4^+$ cells to account for background absorbance resulting from BrdU incorporation of DLD-1 cells.

2 Supplementary Tables

Primer name	Primer sequence
β-actin forward<br>β-actin reverse	5’-GCCAACCGCGAGAAGATGA-3’<br>5’-CATCACGATGCCAGTGGTA-3’
B2M forward<br>B2M reverse	5’-GTGCTCGCGCTACTCTCTC-3’<br>5’-GTCAACTTCAATGTCGGAT-3’
CD40 forward<br>CD40 reverse	5’-AATACTGCGACCCCAACCTA-3’<br>5’-ACAGCTTGTCCAAGGGTGAC-3’
CD58 forward<br>CD58 reverse	5’-GCTTGAACTTAGGGCTGCTTG-3’<br>5’-TTTAAAGGCACATTGCTTGGTA-3’
CD74 forward<br>CD74 reverse	5’-CGCGACCTTATCTCCAACAA-3’<br>5’-GCAGAGTCACCAGGATGGAA-3’
CD80 forward<br>CD80 reverse	5’-CTTCAACTGGAATACAACCAAGC-3’<br>5’-ACAGGGCGTACACTTTCCCT-3’
CD86 forward<br>CD86 reverse	5’-GTGCTTGCTAACTTCAGTCAACCT-3’<br>5’-GCTCGTAACATCAGGGAATGA-3’
CD273 forward<br>CD273 reverse	5’-TGGCAAGTCCTCATATCAAATACA-3’<br>5’-CAAAGTTGCATTCCAGGGTC-3’
CD274 forward<br>CD274 reverse	5’-GAATTGGTCATCCCAGAACTACC-3’<br>5’-TCCAATGCTGGATTACGTCTC-3’
CIITA forward<br>CIITA reverse	5’-GATGCGCTGAGTGAGAACAA-3’<br>5’-CGTCGCAGATGCAGTTATTG-3’
EIF2 forward<br>EIF2 reverse	5’-AACAATTGGCCGCTAAACTTG-3’<br>5’-GATGATGATTCAGAAGCGAGTGT-3’
HLA-A forward<br>HLA-A reverse	5’-GGAGGAGGAAGAGCTCAGATAG-3’<br>5’-ACAGGTCAGCGTGGGAAG-3’
HLA-B forward<br>HLA-B reverse	5’-CCTAGCAGTTGTGGTCATCG-3’<br>5’-ACAGACAGATTCAGAGGCCC-3’
HLA-C forward<br>HLA-C reverse	5’-GCTGTGGTCACCGCTATGAT-3’<br>5’-CTGTCTCAGGCTTTACAAGTGATG-3’
HLA-DMA forward<br>HLA-DMA reverse	5’-TGTGTGGCAAGAAGGTATGG-3’<br>5‘-CGTCGTAGGCCTCAGAGAGT-3’
HLA-DMB forward<br>HLA-DMB reverse	5’-CTCTCACAGCACCTCAACCA-3’<br>5’-TTGGCTACTTGCACAGATGG-3’
HLA-DOA forward<br>HLA-DOA reverse	5’-TGTCCAGTGTCCCCAGGTAAT-3’<br>5’-GACCCGTAGGACAGATGTTGA-3’
HLA-DOB forward<br>HLA-DOB reverse	5’-GTGAATCTGACCCGACTGG-3’<br>5’-ATCTGACCACAAACTGCACCT-3’
HLA-DPA1 forward<br>HLA-DPA1 reverse	5’-GACAGAATGTTCCATATCAGAGC-3’<br>5’-CCTTCTTGTCCAGATCCACATA-3’
HLA-DPB forward<br>HLA-DPB reverse	5’-TTCTGCCCGGAGTAAGACAT-3’<br>5’-GCTCAGGAACCCTGTTTATGC-3’
HLA-DQA forward<br>HLA-DQA reverse	5’-CCCTGTGGAGGTGAAGACAT-3’<br>5’-GGAACCTCATTGGTAGCAGC-3’
HLA-DQB forward<br>HLA-DQB reverse	5’-TGCTTTTCCCTTCGTCTCAG-3’<br>5’-GCGCGTACTCCTCTCGGT-3’
HLA-DRA forward<br>HLA-DRA reverse	5’-CAACTGAGGACGTTTACGACTG-3’<br>5’-TTTGTAAAGCCATTAAAGCAGAAG-3’
HLA-DRB3 forward	5’-TGACACTGATGGTGCTGAGC-3’

Primer name	Primer sequence
HLA-DRB3 reverse	5'-CTGCAGTAATTGTCCACCCG-3'
HLA-E forward<br>HLA-E reverse	5'-GAGCTCAGGTGGAAAAGGA-3'<br>5'-ACAGACAGATTCAGAGGCCC-3'
HLA-F var1 forward<br>HLA-F var1 reverse	5'-GGAGGAAGAAGAGCTCAGATAGAA-3'<br>5'-AATATCCTTGGAAGAGCACCC-3'
HLA-F var2 forward<br>HLA-F var2 reverse	5'-GAGCTCAGATAGAAACAGAGGGA-3'<br>5'-CAAGTGCAATTCTGCTACATTGA-3'
ICAM forward<br>ICAM reverse	5'-CCATCTACAGCTTTCCGGC-3'<br>5'-GACAATCCCTCTCGTCCAGT-3'
ICOSL forward<br>ICOSL reverse	5'-CAGACAGGAAATGACATCGGAG-3'<br>5'-AGTGAGCTCTGTCTCCGGACT-3'
IRF1 forward<br>IRF1 reverse	5'-ACCTCTGAAGCTACAACAGATGA-3'<br>5'-CTGGCTCCTCCTTACAGCTAAA-3'
IRF2 forward<br>IRF2 reverse	5'-ATGCAGAAAGCGAAACGACT-3'<br>5'-CTGTTGTAAGGCACCGGATT-3'
PML forward<br>PML reverse	5'-GGTGTACCGGCAGATTGTG-3'<br>5'-GTAGATGCTGGTCAGCGTAGG-3'
PSMB10 forward<br>PSMB10 reverse	5'-GGGCTTCTCCTTCGAGAACT-3'<br>5'-CAGCCCCACAGCAGTAGATT-3'
PSMB8 forward<br>PSMB8 reverse	5'-TGGGTCCTACATTAGTGCCTTAC-3'<br>5'-GTCCCATGTTCATCCACGTA-3'
PSMB9 forward<br>PSMB9 reverse	5'-ACGTGAAGGAGGTCAGGTATATG-3'<br>5'-ATGACTCGATGGTCCACACC-3'
PSME1 forward<br>PSME1 reverse	5'-CGCTTTCGCTTTCCCTTC-3'<br>5'-TGCATCCAGCTCAGAAATCTT-3'
PSME2 forward<br>PSME2 reverse	5'-GCAAACAGGTGGAGGTCTTC-3'<br>5'-GTCAGCCACATTGAGGGAGT-3'
PSME3 forward<br>PSME3 reverse	5'-CTTCCAAGGAACCAAGGTGTTT-3'<br>5'-GGCGTGTTACATTTCTCAATCA-3'
RPL13A forward<br>RPL13A reverse	5'-GTCGTGCGTCTGAAGCCTAC-3'<br>5'-AGGAGTCCGTGGGTCTTGAG-3'
STAT1 forward<br>STAT1 reverse	5'-TCGGCAGCAGCTTAAAAAGT-3'<br>5'-CACCACAAACGAGCTCTGAA-3'
STAT3 forward<br>STAT3 reverse	5'-AGCTGCACCTGATCACCTTT-3'<br>5'-AATTGGGGGCTTGGTAAAAA-3'
STAT6 forward<br>STAT6 reverse	5'-TTGGCTTCATCAGCAAACAG-3'<br>5'-GGTCCCTTTCCACGGTCA-3'
TAP1 forward<br>TAP1 reverse	5'-ATTCTCACCATAGCCAGTGCAG-3'<br>5'-AGAATCACTCAGGGTGGACG-3'
TAP2 forward<br>TAP2 reverse	5'-GATCTACCAGGAGAGCGTGG-3'<br>5'-GTAAACGTCAGCCCCTTGAG-3'
EMSA probe CIITA-bio*	5'-CCACTTCTGATAAAGCACGTGGTGGC-3'
EMSA probe IRF1-bio*	5'- CAGCCTGATTTCCCCGAAATGACGG-3'

Supplemental Table 1. Oligonucleotide sequences used for qPCR and EMSA, respectively.
* For EMSA probes, only sense orientation is given. Probes were biotin-labeled at the 3'-end.

3 Supplementary figure legend

Figure S1. IL-27 induces expression of CIITA and IRF1 mRNA and protein in the intestinal epithelial cell line DLD-1 as well as in primary epithelial cells. (A, B) Quantitative PCR shows a time-dependent upregulation of (A) CIITA and (B) IRF1 mRNA expression in DLD-1 cells (black bars) and primary IEC (grey bars) through IL-27 stimulation. mRNA expression was normalized to expression in the respective unstimulated cells which was set to 1.0. Data are presented as mean +/- SEM from three independent experiments with 3 samples per group. (C-F) Western Blot experiments with protein isolated from IL-27-stimulated DLD-1 cells (C, D) and primary IEC (E, F) demonstrate increased CIITA and IRF1 protein levels compared to unstimulated cells. While CIITA was absent in unstimulated cells, IRF1 was expressed on basal level. Blots are representative of three independent experiments.

Figure S2. IL-27 induces similar MHCII gene expression levels as IFN-γ and acts synergistically with TNF-α to induce CD74 in IEC. (A) CIITA, HLA-DRA and CD74 mRNA expression levels are slightly higher following stimulation with 50 ng/ml IFN-γ compared to 50 ng/ml IL-27. Data are normalized to the expression induced by IL-27 for each gene. * $p<0.05$ vs. IL-27 (B) IL-27-induced CIITA, HLA-DRA and CD74 protein levels are similar to that induced by IFN-γ. (C) Combined treatment of IEC with IL-27 and TNF-α induces synergistic expression of CD74 protein in IEC as analyzed by western blot in cells stimulated for 48 h. IFN-γ was used as positive control.

Figure S3. IL-27-induces mRNA and protein expression of costimulatory molecules in IEC. (A) Stimulation of DLD-1 cells with IL-27 for different time intervals reveals mRNA upregulation of costimulatory molecules CD40, CD80, CD54 (ICAM1), CD273 (PD-L2), CD274 (PD-L1), and ICOSL, as determined by qPCR. Expression in unstimulated cells was set to 1.0 for each gene and is indicated by the red line. Data are presented as mean ± SEM from two independent experiment, each stimulation performed in triplicates, qPCR in duplicates per sample (B) Western Blot analysis with a specific antibody against ICAM1 confirmed upregulation of the respective protein.

Figure S4. IL-27-induced CIITA and MHC class II expression depends on STAT1, CIITA, and IRF1. (A, B) Immunofluorescence staining of IL-27-stimulated DLD-1 cells confirms upregulation of (A) HLA-DRA and (B) CD74 protein. (C) DLD-1 cells were transfected with siRNA targeting STAT1, STAT3, STAT6, IRF1 or CIITA prior to IL-27 stimulation. Quantitative PCR of IL-27-stimulated DLD-1 cells revealed that the expression of CIITA (left panel), CD74 (middle panel) and HLA-DRA (right panel) depend on the presence of STAT1, IRF1, and CIITA. * $p<0.05$ vs. control+IL-27 as determined by the two-tailed Student's t-test

Figure S5. IEC are able to take up and to process the extracellular antigens ovalbumin and dextran. DLD-1 cells were incubated in 96 well plates with the AlexaFluor 488-labelled antigen ovalbumin (A) or dextran (B) for time intervals as indicated at 37°C (black bars). The intracellular fluorescence (RFU) was measured in a microplate reader. Control reactions were performed at 4°C (grey bars).

Figure S6. IEC are able to phagocytose apoptotic cells. (A) HT-29 cells were treated with a combination of TNF-α and the Smac mimetic SM-164 to induce apoptotic cell death. Immunofluorescence was performed with DAPI-stained nucleus to confirm the typical apoptotic morphology such as cell shrinkage, nuclear condensation, and fragmentation. (B) Alive DLD-1 cells (stained in green) were incubated with apoptotic HT-29 cells (stained in red) for 24h at a ratio of 1:4. Cells were fixed and the nucleus was stained with DAPI (blue). (C)

Alive DLD-1 cells (stained in green) were incubated with apoptotic DLD-1 cells (stained in red) for 24h at a ratio of 1:4. Cells were fixed and the nucleus was stained with DAPI (blue). (D) Alive DLD-1 cells (stained in green) were incubated with necroptotic DLD-1 cells (stained in red) for 24h at a ratio of 1:4. Cells were fixed and the nucleus was stained with DAPI (blue). All imaging was performed by confocal LSM: The right panel of Figure B-D represents a detail of the left picture. (E) Alive DLD-1 cells (stained in green) were either left unstimulated or were stimulated with 50 ng/ml IL-27 for 48h. Then they were incubated with necroptotic HT-29 cells (stained in red) for 24h at a ratio of 1:4. Cells were fixed and the nucleus was stained with DAPI (blue).

Figure S7. The increase in IL-27-mediated immunoproteasome activity depends on the presence of IRF1. (A) The expression of the constitutive protesome subunits PSMB5, 6, and 7 is not influenced by IL-27. qPCR data are normalized to the respective expression in unstimulated cells for each gene. (B, C) DLD-1 cells were transfected with siRNA targeting STAT1, STAT3, IRF1 or CIITA, respectively, prior to IL-27 stimulation. Extracts of these cells were incubated with the quenched fluorescent substrates (Ac-ANW)2R110 (B) or (Ac-PAL)2R110 (C), and fluorescence (indicating substrate processing) was measured in real-time on a microplate reader. The left panels shows the realtime fluorescence data while the right panels represents the relative increase in fluorescence at the timepoint t=24h, compared to the unstimulated control. Data are presented as mean ± SD and are representative from one out three experiment, each performed in biological and technical triplicates. p-values were calculated with the two-tailed Student's t-test.

Figure S8. Overview of the workflow for the coculture experiments of DLD-1 cells and PBMC or $CD4^+$ T cells.

Figure S9. Graphical abstract depicting the results achieved in this study.

Supplementary Figure S1

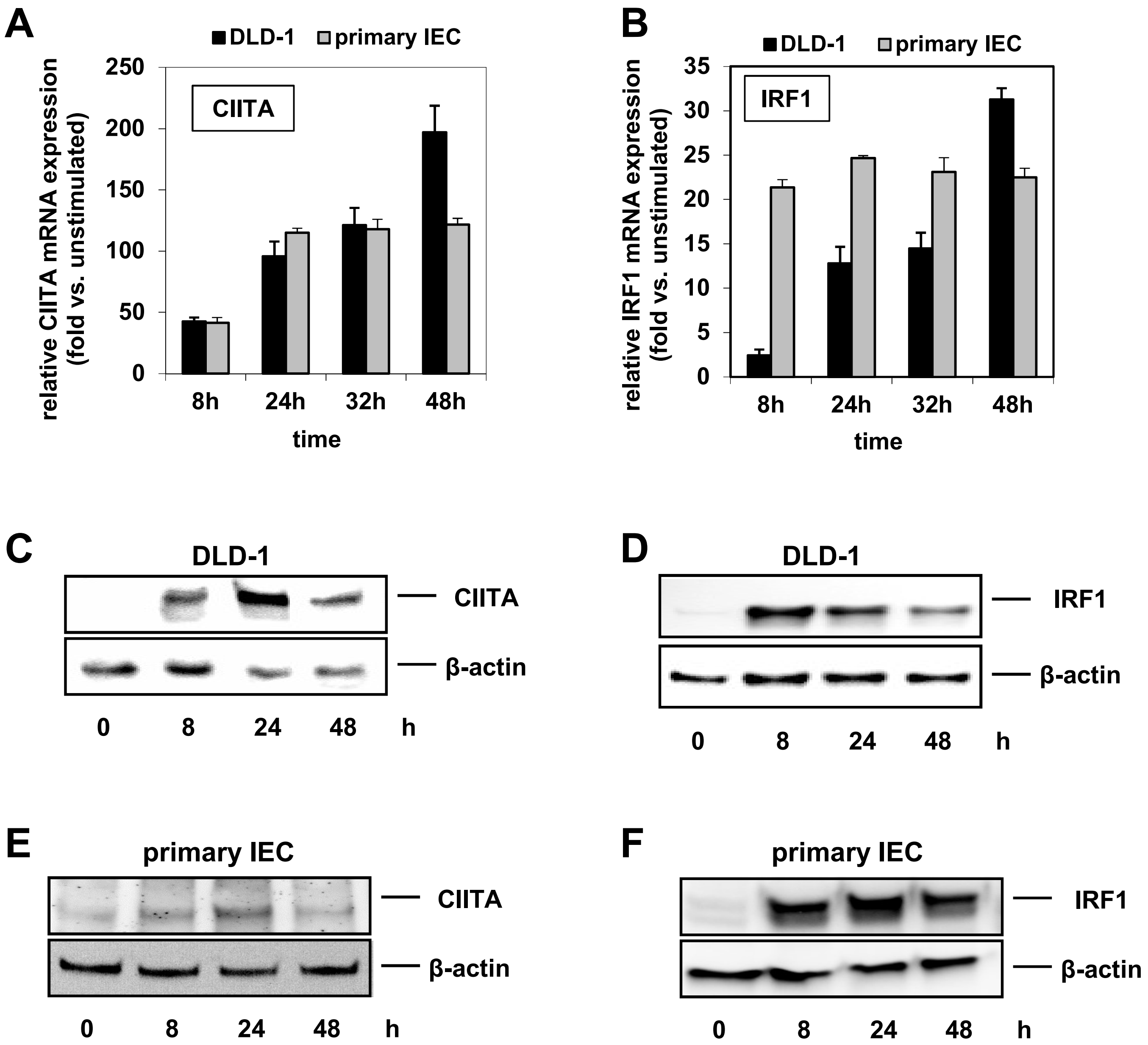

Supplementary Figure S2

A

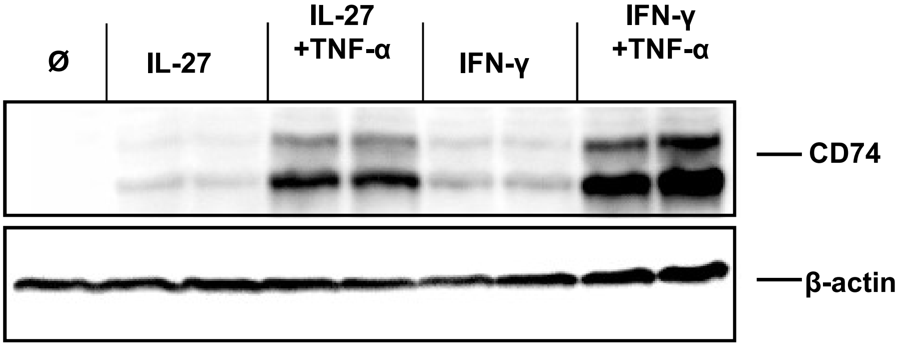

Supplementary Figure S3

A

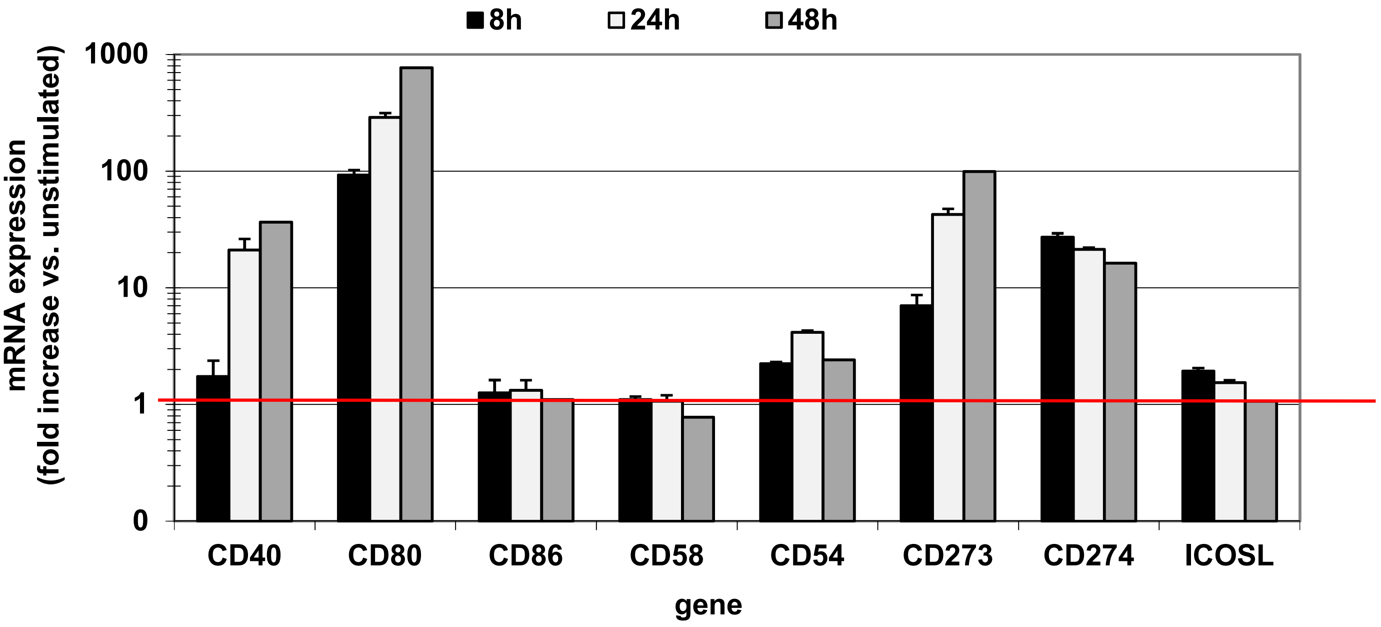

B

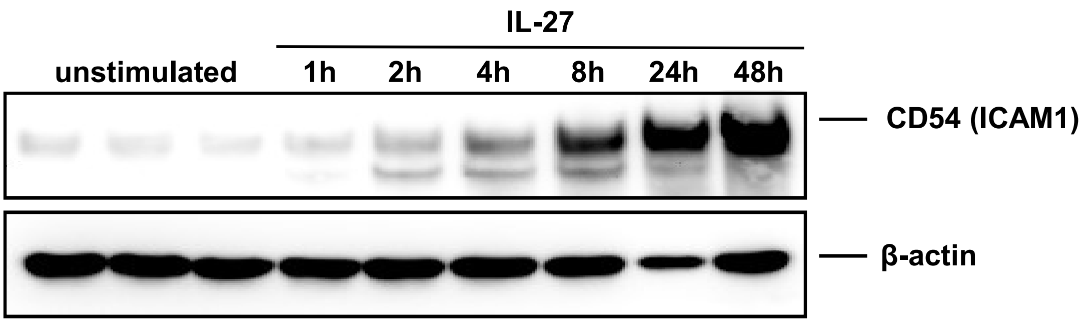

Supplementary Figure S4

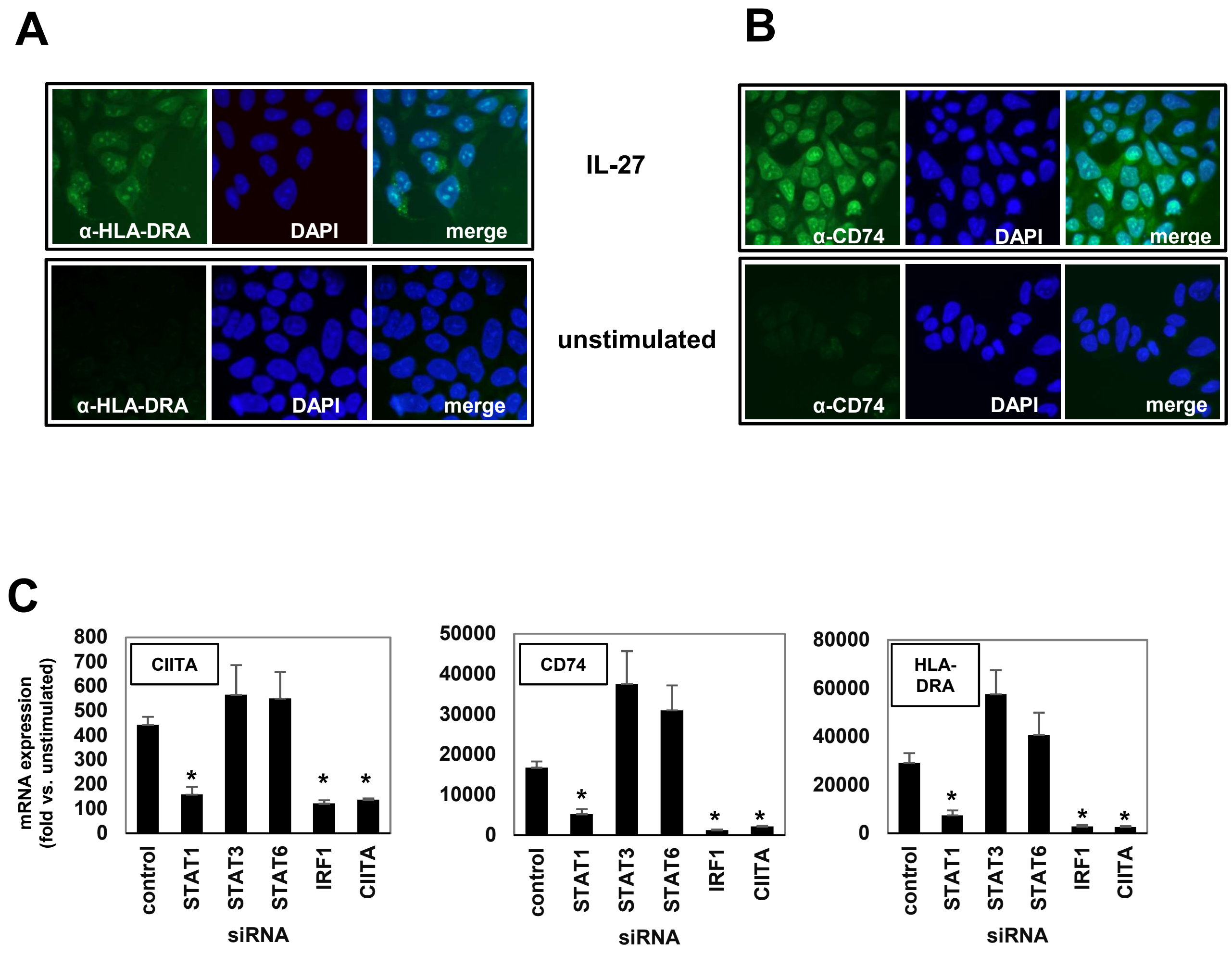

Supplementary Figure S5

A

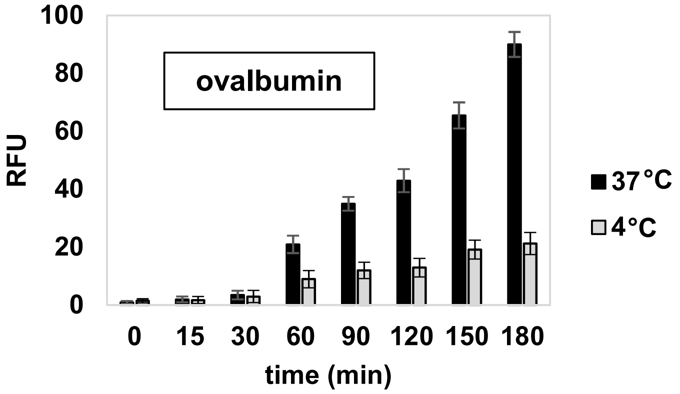

B

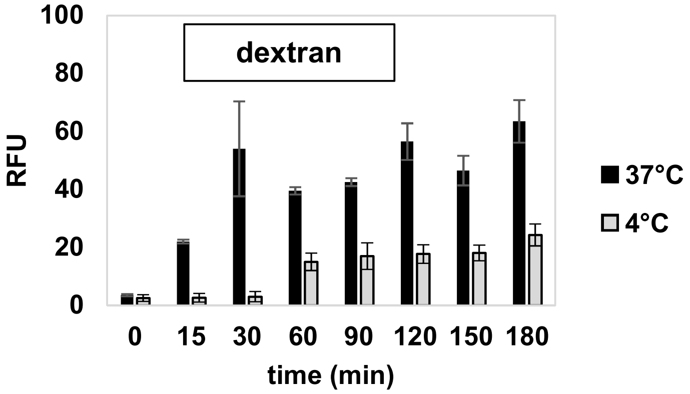

Supplementary Figure S6

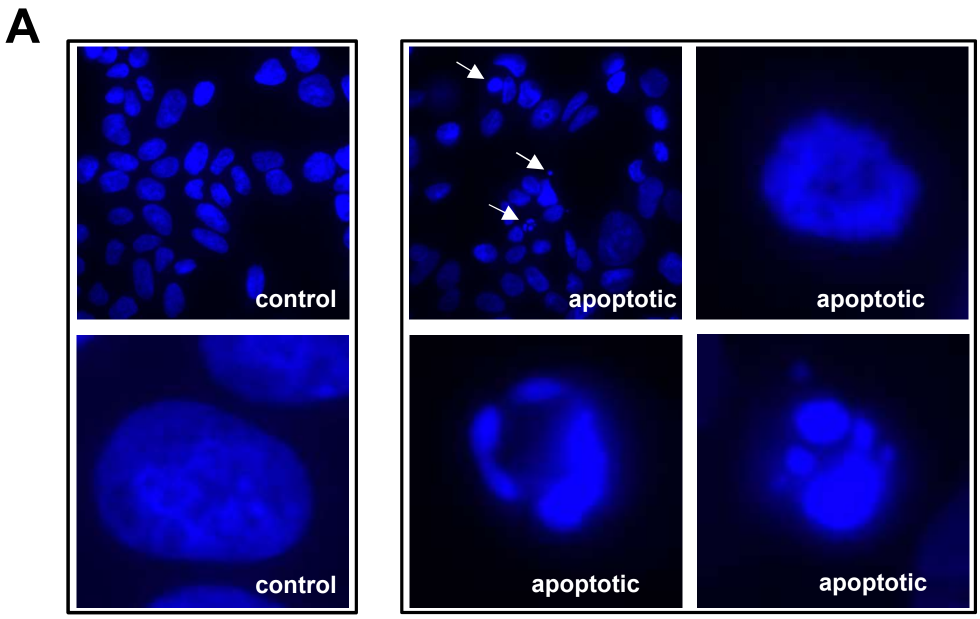

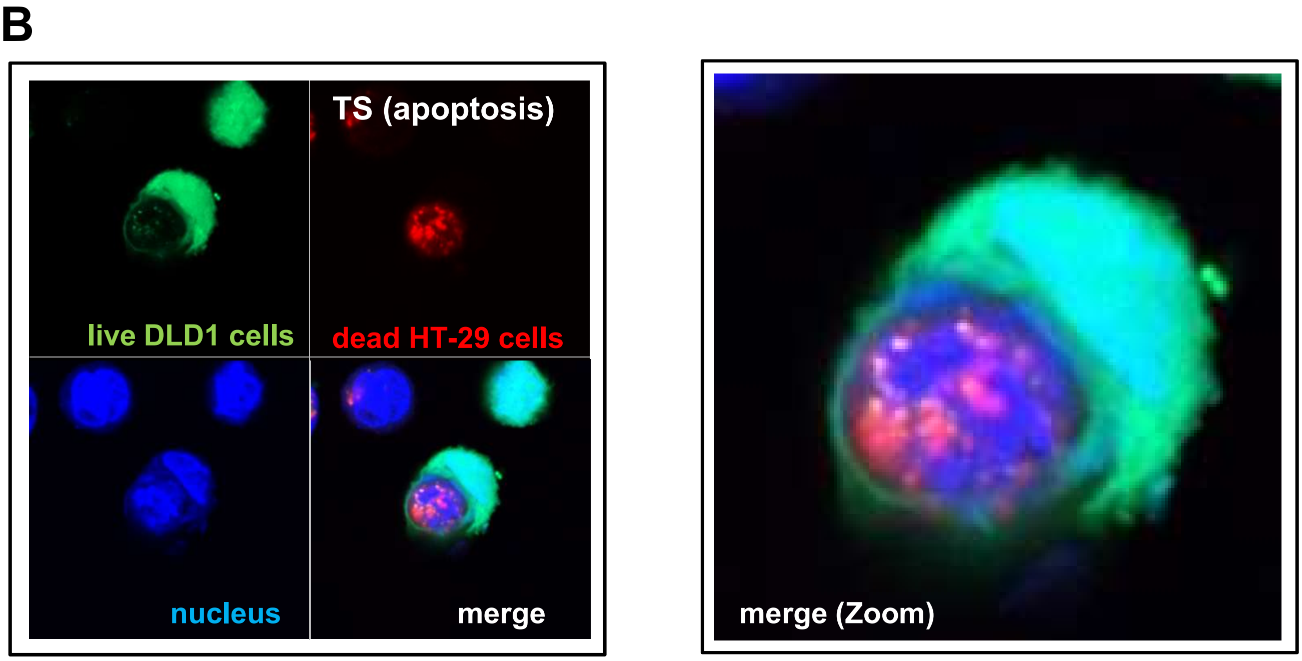

Supplementary Figure S6

C

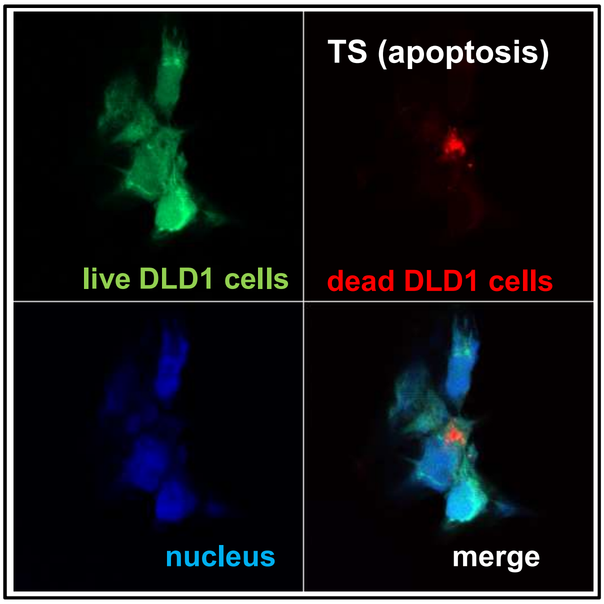

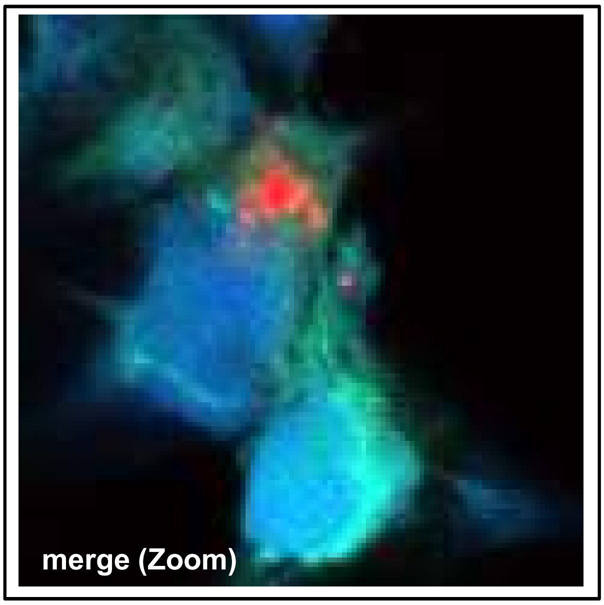

D

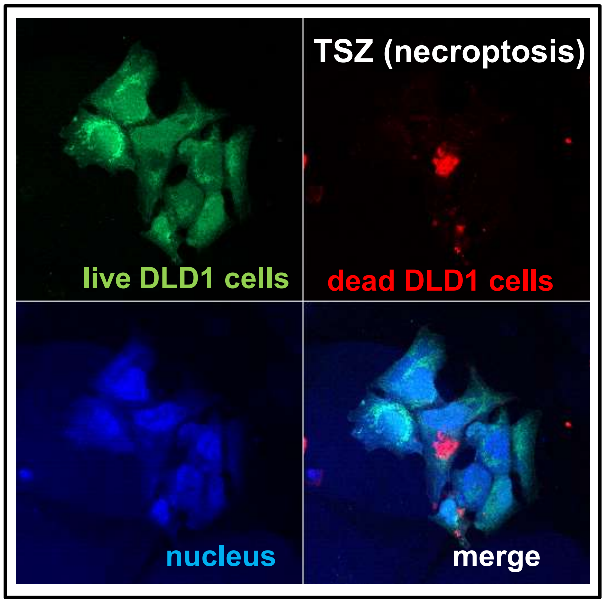

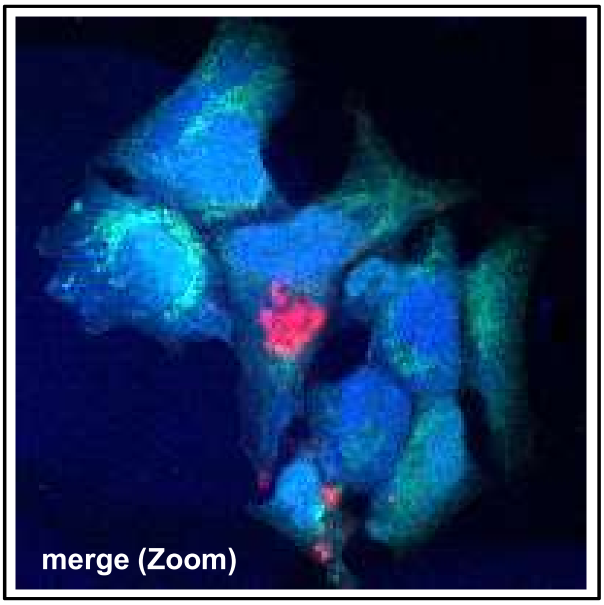

Supplementary Figure S6

E

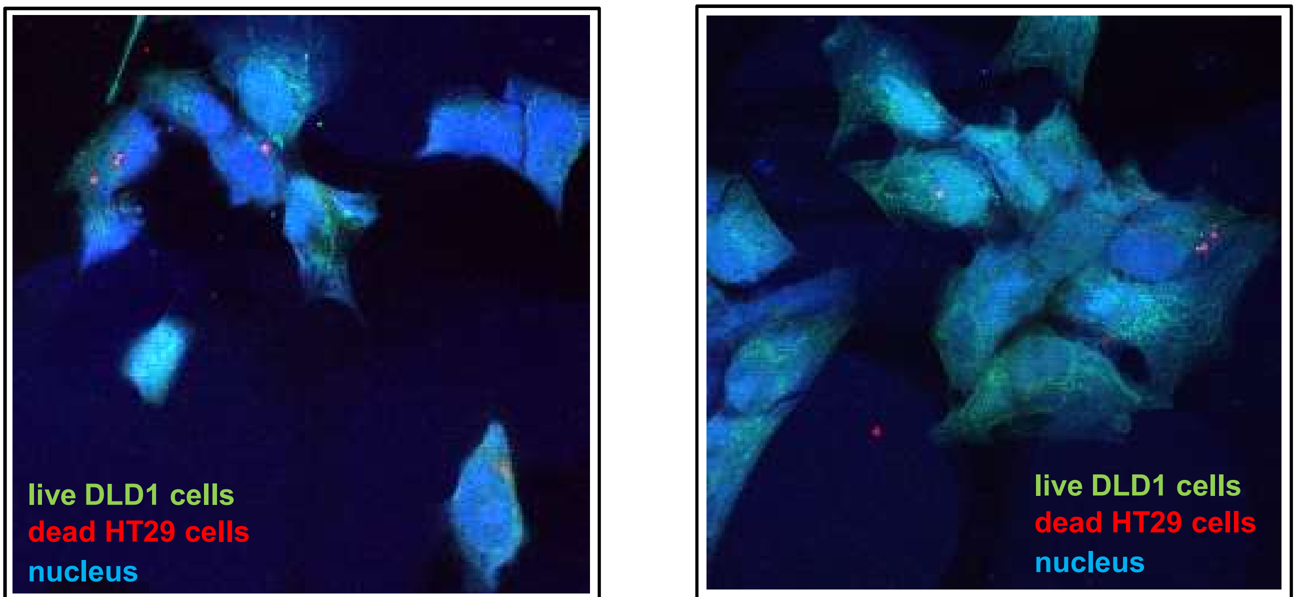

unstimulated

IL-27-stimulated

Supplementary Figure S7

A

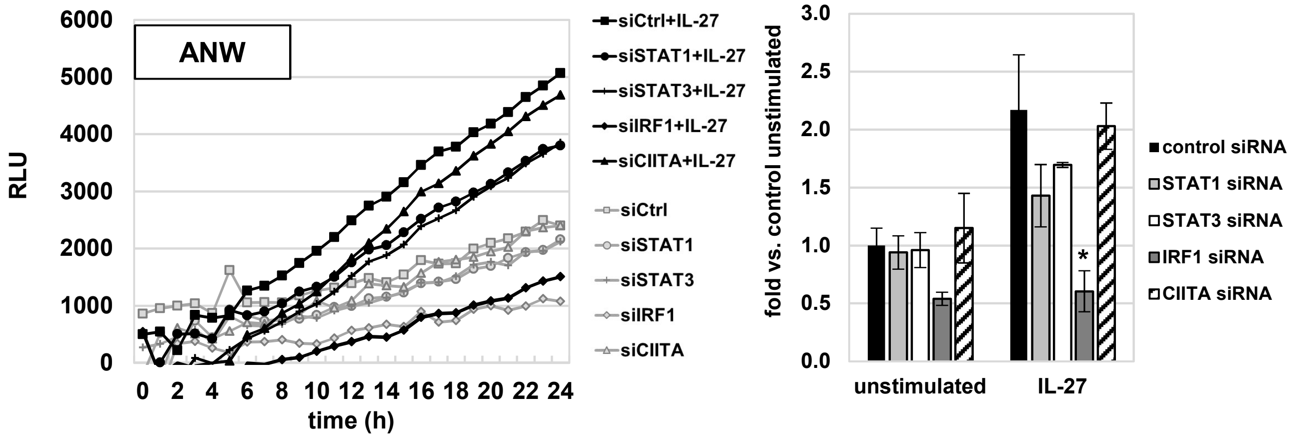

B

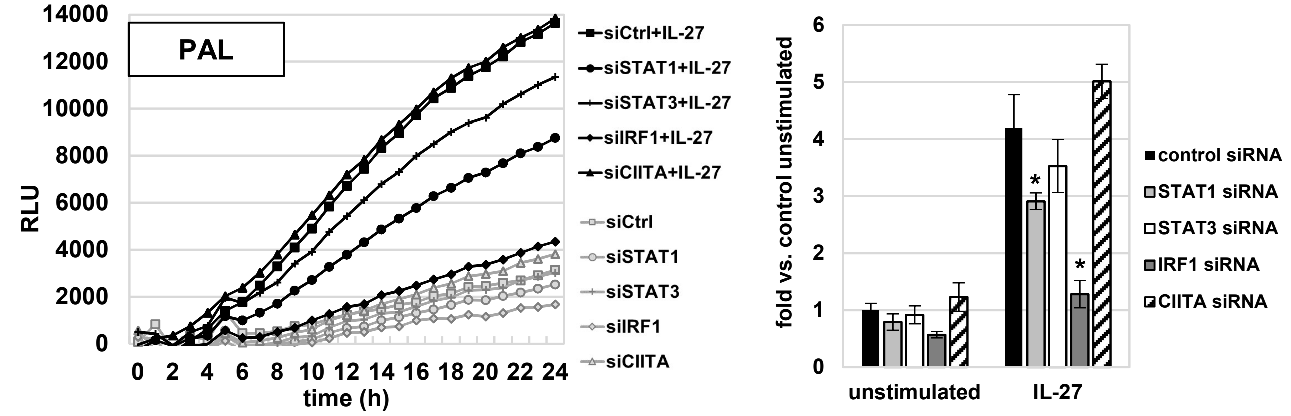

Supplementary Figure S8

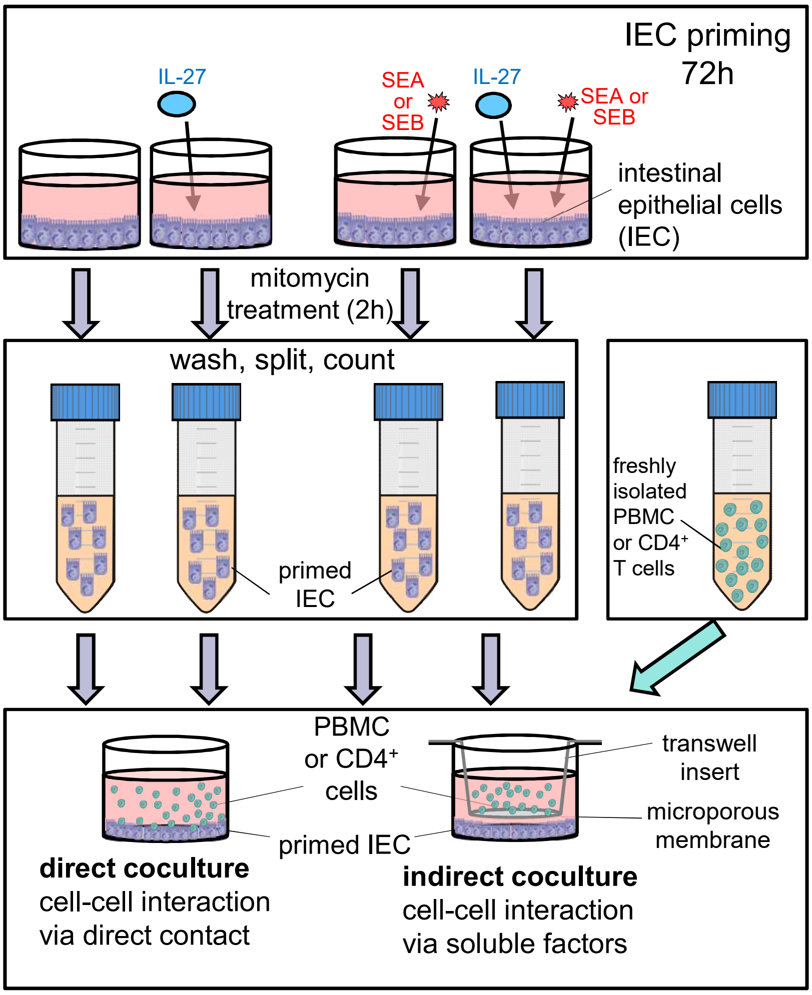

Supplementary Figure S9

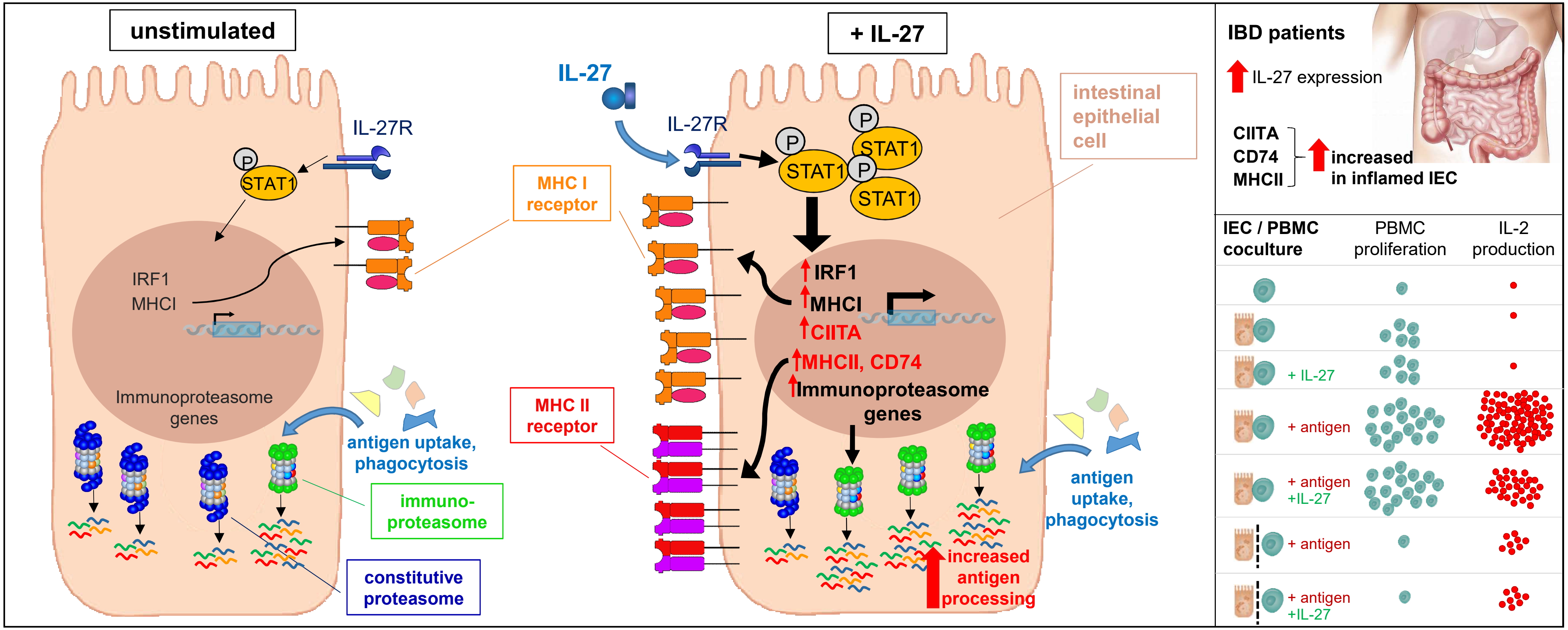

Supplement: Supplementary file 1 [file DataSheet_1.pdf]
